# Turn-On Fluorescent pH Probes for Monitoring Alkaline pHs Using Bis[2-(2′-hydroxyphenyl)benzazole] Derivatives

**DOI:** 10.3390/s23042044

**Published:** 2023-02-11

**Authors:** Hyuna Lee, Suji Lee, Min Su Han

**Affiliations:** Department of Chemistry, Gwangju Institute of Science and Technology (GIST), 123 Cheomdangwagi-ro, Buk-gu, Gwangju 61005, Republic of Korea

**Keywords:** fluorescent probe, bis[2-(2′-hydroxyphenyl)benzazole] derivatives, alkaline pH probe

## Abstract

For surveilling human health, industries, and the environment, pH monitoring is important. Numerous studies on fluorescent probes have been conducted to monitor various pH ranges. However, fluorescent probes that are capable of sensing alkaline regions are rare. In this study, we propose turn-on-type fluorescent probes for detecting alkaline pHs using bis[2-(2′-hydroxyphenyl)benzazole] (bis(HBX)) derivatives. These probes have high p*K*_a_ values (from 9.7 to 10.8) and exhibit strong fluorescence intensity and color changes at alkaline pHs. Probes derived from bis(HBX) exhibit good photostability, reversibility, and anti-interference toward pH variations, which can be identified as a certain fluorescence change toward a basic pH. Therefore, compounds would be advantageous to use fluorescent probes for monitoring alkaline pH changes.

## 1. Introduction

Accurate monitoring of pH is valuable for human health, diseases, industry, and the environment [1]. Therefore, various methods have been developed to monitor pH, such as those utilizing microelectrodes, nuclear magnetic resonance (NMR), colorimetry, and fluorescence [2,3,4,5,6]. Compared to other pH measurement methods, fluorescence technology is beneficial because of its easy visualization and operation, nondestructive character, high sensitivity and selectivity, and the ease of availability of a wide range of dyes as pH indicators [7,8,9,10].

Because of these advantages, numerous fluorescent probes have been developed to monitor pH for various applications, including biological, medicinal, and biotechnological applications [11,12,13]. Commonly known fluorophores are tetraphenylethene, coumarin, naphthalimide, rhodamine, boron dipyrromethene (BODIPY), cyanine (Cy) [14], and 2-(2-hydroxyphenyl)benzothiazole [15]. Organic fluorescent pH probes commonly have a characteristic nitrogen- or oxygen-containing group where protonation or deprotonation occurs depending on the pH, changing the fluorescence of the fluorophore [14,16]. Using this property, several researchers have developed fluorescent probes that can monitor various pH ranges.

Although the developed fluorescent pH probes have been successful in monitoring acidic to near-neutral pH conditions [17,18,19,20], probes capable of monitoring alkaline regions are still rare. pH detection in the alkaline range is valuable in several fields, such as leather processing, wastewater treatment, the paper industry, nuclear fuel reprocessing, agriculture, detection of corrosion of steel-reinforced concrete structures, and metal mining and finishing [21,22]. Therefore, several fluorescent pH probes for detecting alkaline pHs have been developed, such as perylene tetra-(alkoxycarbonyl) derivative-based [21], BODIPY-based [22], and coumarin-based [23] alkaline pH fluorescent probes. However, most fluorescent probes reported to monitor pHs above 10 are turn-off type, in which fluorescence intensity decreases in the alkaline pH [4,11,21]. Fluorescent probes with turn-on signals are generally considered more efficient. Compared with “turn-off” probes, the main advantage of “turn-on” probes is that they can easily measure low-concentration contrasts against a background, which increases sensitivity [24]. Therefore, it is particularly meaningful to develop new turn-on-type fluorescent probes that can detect an extremely alkaline pH.

**Scheme 1:**
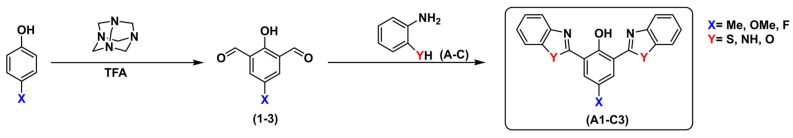
Two–step synthesis of bis(HBX) derivatives.

In this study, we synthesized bis[2-(2′-hydroxyphenyl)benzazole] (bis(HBX)) derivatives that can be used in monitoring alkaline pHs. HBX-based probes exhibit a significant Stokes shift because of their intramolecular hydrogen-bonding properties, which exhibit excited-state intramolecular proton transfer (ESIPT) [25,26]. Owing to this property, HBX derivatives have been developed as fluorescent pH probes. However, the reported pH probes using mono HBX monitored a limited range of pHs (from acidic to near neutral) [1,27,28]. Herein, we envisioned that this limitation could be solved by combining the two HBX moieties into one molecule. According to studies, molecules that combine two benzazole groups have extra proton-binding sites and more effective ESIPT than mono HBX [25]. The intramolecular hydrogen bonding energies were found to be around 10 kcal/mol [29,30]. Unlike the previous mono HBX, bis(HBX) derivatives provide two intramolecular hydrogen-bonding acceptors, as shown in Scheme 1. The hydrogen bond can increase the deprotonation energy of the OH group in bis(HBX) derivatives [31,32]. Because of these characteristics, it is also expected that bis(HBX) derivatives should exhibit higher p*K*_a_ values than mono HBX and interesting fluorescence patterns under extreme basic conditions. In addition, we speculate that the properties of the synthesized molecules could be tuned by changing the benzazole and functional groups bound to the central phenol. Consequently, nine bis(HBX) derivatives with two benzazole groups and various functional groups attached to the central phenol were synthesized, and the fluorescence change was measured according to the pH. Among the synthesized nine compounds, three compounds (**A1**, **A2**, and **C1**) with better properties were selected as alkaline pH probes. Probes exhibited the characteristics of “turn-on” and color change in the probe solution from colorless to yellow when the pH value increased from acidic to basic. In addition, unlike other probes that have a sigmoid-type pH titration plot, **Al**, **A2**, and **C1** show a pH titration plot in the form of a step function with a relatively narrow transition range, which has the advantage of clearly checking the pH before and after p*K*_a_.

## 2. Materials and Methods

### 2.1. Materials and Instruments

Chemical reagents were purchased from commercial sources (Sigma-Aldrich (St. Louis, MO, USA), Alfa Aesar (Ward Hill, MA, USA), Duksan Pure Chemicals, Daejung Chemicals, Samchun Pure Chemical, and Tokyo Chemical Industry) and used without further purification. ^1^H-NMR and ^13^C-NMR spectra were recorded on an NMR spectrometer (400 MHz, JEOL and Bruker). Mass spectra were obtained using a mass spectrometer (LTQ Orbitrap, Thermo Fisher Scientific, Waltham, MA, USA). The melting points of the materials were determined using melting point determination apparatus (M-565, Buchi, Meierseggstrasse 40, 9230 Flawil, Switzerland). Ultraviolet–visible (UV-vis) and fluorescence spectra, which were used for screening the materials, were recorded using UV-vis (V-630, JASCO Inc., Easton, MD, USA) and fluorescence spectrophotometers (Cary Eclipse, Agilent, Santa Clara, CA, USA), respectively. A pH meter (SevenCompact S210, Mettler Toledo, Greifensee, Switzerland) and a pH electrode (InLab Science Pro-ISM, Metter Toledo, Greifensee, Switzerland) were used to perform pH measurements.

### 2.2. Synthesis of bis(HBX) Derivatives (***A1***, ***A2***, and ***C1***)

#### 2.2.1. General Procedure of Bis-Benzothiazolyl Phenol Derivatives (**A1**, **A2**) [33]

The synthesis of bis-benzothiazolyl phenol derivatives is shown in Scheme S2. To a solution of 2-hydroxyisophthalaldehyde derivatives (3.0 mmol) and 2-aminothiophenol (0.963 mL, 9.0 mmol) in 20 mL of EtOH, HCl (35.0%, 0.278 mL, 9.0 mmol) and H_2_O_2_ (35.0%, 0.367 mL, 12.0 mmol) were added dropwise, and the mixture was stirred for 1 h at 0 °C. After completion of the reaction, the mixture was filtered, washed with EtOH, and dried under a vacuum. The crude mixture was purified by column chromatography using DCM on silica gel.

The compound 2,6-bis(2-benzothiazolyl)-4-methylphenol (**A1**) was obtained as a yellow solid (0.702 g, 62.5%). m.p: 253.8–257.1 °C; ^1^H-NMR (400 MHz, CDCl_3_) δ 13.97 (s, 1H), 8.08 (d, *J* = 8.2 Hz, 4H), 7.96 (d, *J* = 7.9 Hz, 2H), 7.55–7.51 (m, 2H), 7.45–7.41 (m, 2H), 2.48 (s, 3H); ^13^C-NMR (101 MHz, CDCl_3_) δ 154.2, 151.7, 131.8, 129.0, 126.4, 125.2, 122.4, 121.5, 20.5; HRMS (ESI): *m*/*z* calcd for C_21_H_13_N_2_OS_2_ [M–H]^−^ 373.0474, found 373.0474.

The compound 2,6-bis(2-benzothiazolyl)-4-methoxyphenol (**A2**) was obtained as a yellow solid (0.470 g, 40.1%). m.p: 228.0–232.3 °C; ^1^H-NMR (400 MHz, CDCl_3_) δ 13.70 (s, 1H), 8.08 (d, *J* = 7.9 Hz, 2H), 7.96 (d, *J* = 7.9 Hz, 2H), 7.85 (s, 2H), 7.54 (t, *J* = 7.0 Hz, 2H), 7.43 (t, *J* = 7.6 Hz, 2H), 3.98 (s, 3H); ^13^C-NMR (101 MHz, CDCl_3_) δ 152.4, 151.0, 134.2, 126.8, 125.7, 122.4, 121.6, 119.8, 117.2, 56.6; HRMS (ESI): *m*/*z* calcd for C_21_H_13_N_2_O_2_S_2_ [M–H]^−^ 389.0423, found 389.0423.

#### 2.2.2. General Procedure of Bis-Benzoxazolyl Phenol Derivatives (**C1**) [34]

The synthesis of bis-benzoxazolyl phenol derivatives is shown in Scheme S2. A mixture of 2-hydroxyisophthalaldehyde derivatives (3.0 mmol), 2-aminophenol (0.655 g, 6.0 mmol), and phenylboronic acid (0.109 g, 0.9 mmol) in 30 mL of MeOH was magnetically stirred for 5 min. Sodium cyanide (0.192 g, 4.8 mmol) was added. The mixture was thereafter stirred for 18 h at 25 °C. The flask was subsequently immersed in an ice bath. The precipitate was collected via vacuum filtration and washed with cold water. The solid was purified by recrystallization from MeOH/H_2_O.

The compound 2,6-bis(2-benzoxazolyl)-4-methylphenol (**C1**) was obtained as an orange solid (0.569 g, 55.4%). m.p: 172.1–175.6 °C; ^1^H-NMR (400 MHz, DMSO-*d*_6_) δ 12.20 (s, 1H), 8.09 (s, 2H), 7.84–7.80 (m, 4H), 7.48–7.41 (m, 4H), 2.40 (s, 3H); ^13^C-NMR (101 MHz, DMSO-*d*_6_) δ 161.8, 155.3, 149.9, 140.6, 133.5, 129.7, 126.5, 125.7, 120.1, 113.7, 111.6, 20.3; HRMS (ESI): *m*/*z* calcd for C_21_H_13_N_2_O_3_ [M–H]^−^ 341.0931, found 341.0923.

### 2.3. Screening of the Bis(HBX) Derivatives

For the determination of the pH, solutions comprising each bis(HBX) derivative (50 μM; 10% DMSO) and Britton–Robinson (B–R) buffers (pH 1.0, 2.0, 3.0, 4.0, 5.0, 6.0, 7.0, 7.5, 8.0, 8.3, 8.7, 9.0, 9.3, 9.7, 10.0, 10.3, 10.5, 10.7, 11.0, 12.0, 13.0, 13.9; 10 mM) were arranged. B–R buffers with various pH values were prepared with H_2_O as the solvent using a known procedure. All spectroscopic experiments were conducted under the following conditions: The UV-vis and fluorescence spectra of the solutions were measured at 25 °C; the photomultiplier tube (PMT) voltage was 400 V; and the excitation and emission slit widths were 5 nm. Each experiment was repeated thrice.

### 2.4. Calculation of the pK_a_ and Quantum Yield (Φ)

The p*K*_a_ value of each bis(HBX) derivative was obtained by fitting the Boltzmann function to the titration data of its fluorescence response to pH. To calculate the quantum yield, coumarin 153 (C-153, Φ_*st*_ = 0.53) in EtOH was used as the standard dye. The slope of each compound was calculated by plotting the absorbance on the x-axis and the integration of the fluorescence intensity on the y-axis. The photoluminescence quantum yield (Φ) was calculated using Equation (1) [35].

(1) $$\Phi =\Phi_{St}\cdot \frac{compound\;slope}{Standard\;slope}\cdot \frac{compound\;solvent\;refractive\;index^{2}}{standard\;solvent\;refractive\;index^{2}}\;$$

### 2.5. Photostability, Ionic Interference, and Reversibility Study

The photostability of the bis(HBX) derivatives (**A1**–**3**, **B1**–**3**, and **C1**–**3**) was analyzed using solutions comprising each bis(HBX) derivative (50 μM; 10% DMF) and B–R buffers (10 mM). The fluorescence spectra were recorded for 30 min. Thereafter, three of the nine compounds were selected and measured three times under the following conditions: bis(HBX) derivatives (**A1**, **A2**, and **C1**: 50 μM; 10% DMSO) and B–R buffers (pH 6.0/12.0 for **A1**, **A2** and pH 6.0/13.0 for **C1**; 10 mM).

To analyze the selectivity of the fluorescence responses of the bis(HBX) derivatives to each pH in the presence of other interfering ionic species, solutions comprising each bis(HBX) derivative (**A1**, **A2**, and **C1**: 50 μM; 10% DMSO) and B–R buffers (pH 6.0/12.0 for **A1**, **A2** and pH 6.0/13.0 for **C1**; 10 mM) with or without various ions (Na^+^: 10 mM; K^+^: 10 mM; Ca^2+^: 1 mM; Mg^2+^: 1 mM; Fe^2+^: 100 μM; Cu^2+^: 100 μM; Zn^2+^: 200 μM; F^−^: 1 mM; Cl^−^: 1 mM; HSO_4_^−^: 1 mM; H_2_PO_4_^−^: 1 mM; NO_3_^−^: 1 mM; SCN^−^: 1 mM for **A1**, **A2** and Na^+^: 10 mM; K^+^: 5 mM; Ca^2+^: 20 μM; Mg^2+^: 10 μM; Fe^2+^: 50 μM; Cu^2+^: 5 μM; Zn^2+^: 5 μM; F^−^: 1 mM; Cl^−^: 1 mM; HSO_4_^−^: 500 μM; H_2_PO_4_^−^: 1 mM; NO_3_^−^: 1 mM; SCN^−^: 1 mM for **C1**) were arranged. The fluorescence spectra were recorded at suitable excitation wavelengths.

The fluorescence reversibility of the bis(HBX) derivatives was analyzed using solutions comprising each bis(HBX) derivative (**A1**, **A2**, and **C1**: 25 μM; 30% DMSO) and B–R buffers (pH 12.0 for **A1**, **A2** and pH 13.0 for **C1**; 10 mM). The fluorescence spectrum of each solution was measured before the pH was fitted through the dropwise addition of HCl and NaOH aqueous solutions. The fluorescence spectrum of the fitted solution was then measured again. The pH decreased and subsequently increased during the four cycles.

The data were collected using an Agilent fluorescence spectrophotometer at 25 °C. Each experiment was repeated thrice. For all measurements, the PMT was 400 V, and the slit width was 5/5 nm.

## 3. Results and Discussion

### 3.1. Synthesis of the Bis(HBX) Derivatives for Fluorescent Probes

The nine compounds were synthesized with benzazole groups (benzothiazole, benzimidazole, and benzoxazole) at the *ortho* position of the central phenol and functional groups (-F, -Me, and -OMe) at the *para* position by simple two-step reactions (Scheme 1). The 2,6-diformyl-phenol derivatives (**1**–**3**) were obtained by the Duff reaction of the phenol derivatives with three equivalents of hexamethylene tetramine using trifluoroacetic acid (TFA) as the solvent (Scheme S1) [36,37,38]. Thereafter, the bis(HBX) derivatives (**A1**–**C3**) were synthesized via the condensation of the 2,6-diformyl-phenol derivatives (**1**–**3**) with the aniline derivatives (**A**–**C**) (Scheme S2). Characterization data, such as NMR results, mass, melting point, and yield, are presented in the Supplementary Materials (Table S1, Figure S8–34).

### 3.2. Spectroscopic Properties of the Bis(HBX) Derivatives

**Figure 1:**
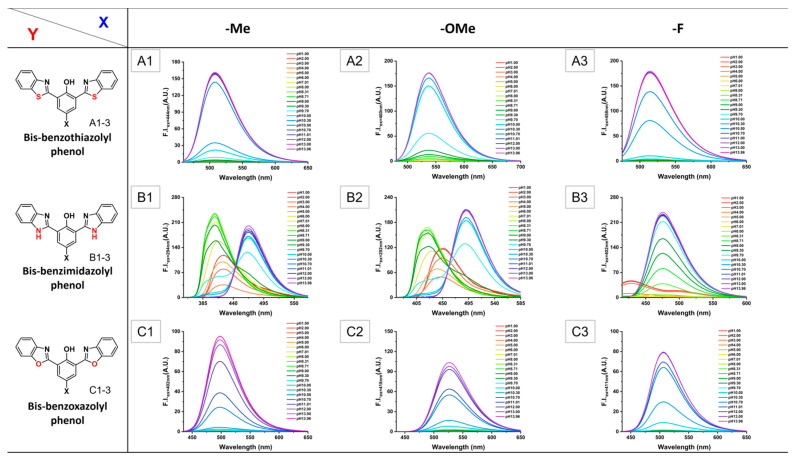
Fluorescence spectra of the nine bis(HBX) derivatives (**A1**–**C3**, pH 1.0–13.9).

The UV-vis and fluorescence responses of the nine bis(HBX) derivatives according to pH changes were investigated under a wide range of pH conditions (pH 1.0–13.9). The UV-vis spectra of the nine compounds exhibited similar patterns, depending on the type of bound benzazole (Figure S1). The fluorescence spectra according to the pH are listed in Figure 1. As we expected, among the synthesized nine bis(HBX) derivatives, the benzothiazole (**A1**–**3**) and benzoxazole (**C1**–**3**) conjugated compounds exhibited strong turn-on fluorescence responses under basic pH conditions in contrast to their HBX mono forms, which exhibited fluorescence changes in acidic to neutral pH ranges [1,27,28]. In contrast, compounds bearing benzimidazole (**B1**–**3**) exhibited unique spectra, showing ratiometric fluorescence responses from pH 1.0 to 13.9.

To figure out the compound showing the best performance as a pH fluorescence probe among the nine bis(HBX) derivatives, we compared these in terms of photostability and solubility. First, photostability was studied by measuring the fluorescence intensity for 30 min in the pH regions, including the transition ranges (Figure S2). While fluorescence changes for compounds **A1**–**3**, **C1**, and **C3** were negligible, compounds **B1**–**3** and **C2** exhibited unstable properties where the emission wavelengths or fluorescence intensity changed over time. The photochemical instability of a fluorescent dye is well correlated with the excited-state lifetime of the fluorescent dye because the fluorescent dye in an excited state can react with its surrounding molecules [39]. The excited state lifetimes of mono HBX were determined to be 2-(2′-hydroxyphenyl)benzothiazole (HBT) (14 ps in ACN) [40], 2-(2′-hydroxyphenyl)benzoxazole (HBO) (1080 ps in DMSO) [41], and 2-(2′-hydroxyphenyl)benzimidazole (HBI) (1.5 ns in EtOH) [42], respectively. Therefore, the differences in the photochemical instabilities of the bis(HBX) derivatives may come from the length of their excited-state lifetimes. Further, the solubility of the compounds was confirmed in a solvent, and **A3** exhibited an extremely low solubility compared with those of the other compounds (Figure S3).

For the four compounds (**A1**, **A2**, **C1**, and **C3**), the p*K*_a_ and quantum yield calculations were performed (Table 1). Various p*K*_a_ values of 9.8 (**A2**), 10.4 (**A1**, **C3**), and 10.8 (**C1**) indicated that the synthesized probes were capable of sensing a wide range of alkaline pHs. Based on the data, **A1**, **A2**, and **C1** with various p*K*_a_ values between 9.7 and 10.8 were selected as fluorescent probes for monitoring alkaline pH ranges, and further studies were conducted (**C3** exhibited a p*K*_a_ value similar to that of **A1** but a smaller quantum yield than **A1**; thus, **A1** was selected).

### 3.3. Fluorescence Response of the Selected Probes (***A1***, ***A2***, and ***C1***) to pH

**Figure 2:**
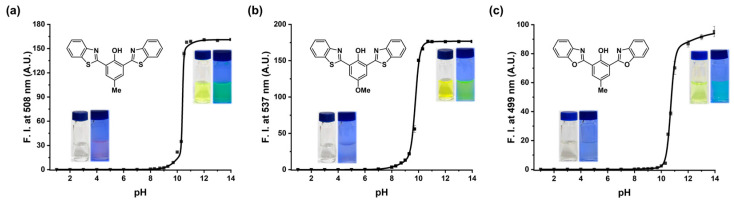
pH titration plots at emission maxima (pH 1.0–13.9). (**a**) Plot of **A1**; λ_ex_ = 444 nm. (**b**) Plot of **A2**; λ_ex_ = 460 nm. (**c**) Plot of **C1**; λ_ex_ = 402 nm.

Figure 2 shows the pH titration plots of the selected probes (**A1**, **A2**, and **C1**). The fluorescence intensity of **A1**, having a p*K*_a_ value of 10.4, was extremely low from pH 1.0 to 9.0, but the fluorescence intensity increased approximately 16-fold for pH 9.7 to 10.7 and saturated above pH 10.7. Similar to the fluorescence response of **A1**, **A2**, having a p*K*_a_ value of 9.8, exhibited an extremely low fluorescence intensity from pH 1.0 to 8.0, and the intensity dramatically increased approximately 12-fold for pH 9.0 to 10.7 and saturated over pH 10.7. The fluorescence intensity of **C1**, with a p*K*_a_ value of 10.8, was extremely low from pH 1.0 to 10.0, exhibiting a fluorescence change that increased approximately 24-fold from pH 10.3 to 13.9.

The probes exhibited an extremely sharp increase in fluorescence intensity with a narrow pH transition range. By having the pH titration plots in the form of a step function, the probes could clearly monitor the pH before and after p*K*_a_. Moreover, the solutions for the three compounds changed from colorless to yellow as the pH increased to the alkaline range, showing that the pH change for the alkaline range could be checked with the naked eye using the probes. The compounds exhibited high p*K*_a_ values and strong turn-on fluorescence signals at alkaline pH values, indicating that these compounds are suitable for monitoring alkaline pHs.

### 3.4. Photostability, Reversibility, Ionic Interference Study, and the Applicability of the Selected Probes

**Figure 3:**
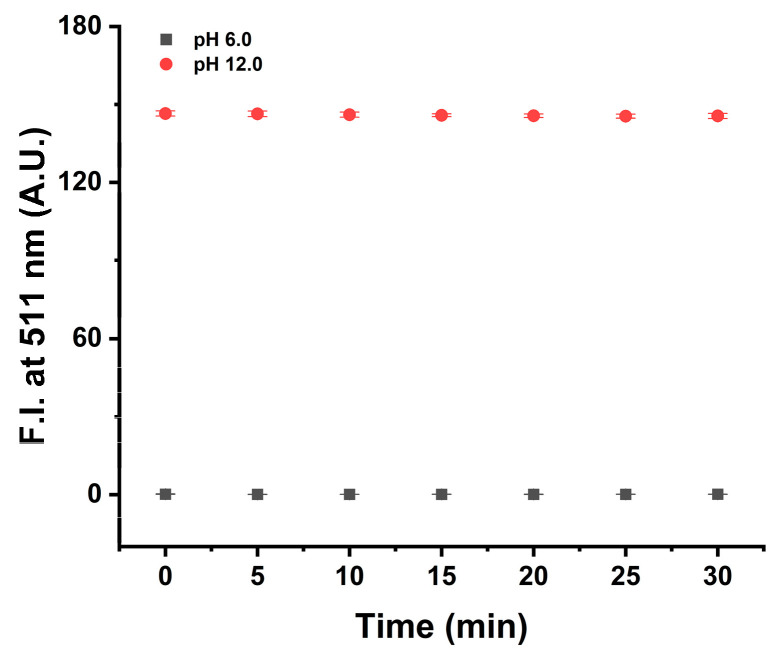
Photostability of **A1** (50 μM) at different pHs (pH 6.0 and 12.0) for 30 min at λ_ex_ = 444 nm.

For the three compounds (**A1**, **A2**, and **C1**) selected as fluorescent probes, in-depth studies were conducted, such as photostability, reversibility, and ionic interference studies, for evaluating the performance of fluorescent probes. To evaluate the photostability of the probes, changes in the fluorescence intensity of the three probes were measured for 30 min at 5 min intervals at each pH that the fluorescence signal turned on and off. As shown in Figure 3, the fluorescence intensity of **A1** remained stable at pH 6.0 and 12.0, and the other probes also exhibited good photostability under acidic and alkaline conditions (Figure S4).

**Figure 4:**
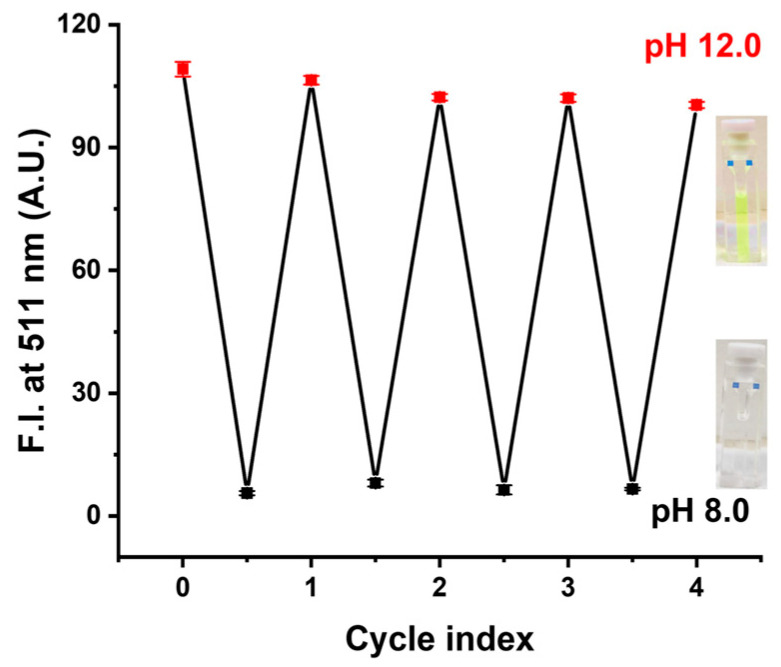
pH reversibility study of **A1** (25 μM) between pH 12.0 and 8.0 for four cycles, λ_ex_ = 444 nm.

The reversibility of the fluorescence response was investigated by changing the pH using aqueous HCl and NaOH solutions. As shown in Figure 4, the reversibility test of **A1** started from pH 12.0 to 8.0, for four cycles. During the cycles, the enhanced fluorescence signal at pH 12.0 was quenched immediately at pH 8.0 by adding HCl solution, exhibiting good reversibility. Simultaneously, the solution changed from yellow to colorless at pH 12.0 and 8.0 under natural light. Similar reversible results were obtained for **A2** and **C1** (Figure S5). These results show that the fluorescence responses of the probes possess a clear on–off switch with pH changes, and the probes have good reversibility.

**Figure 5:**
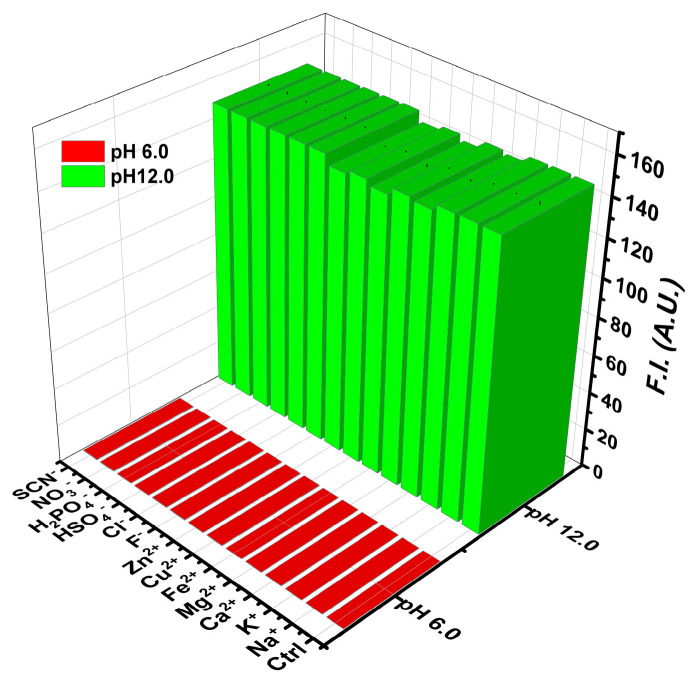
Fluorescence intensity of **A1** (50 μM) at pH 6.0 and 12.0 in the absence (control) and presence of various ionic species (Na^+^: 10 mM; K^+^: 10 mM; Ca^2+^: 1 mM; Mg^2+^: 1 mM; Fe^2+^: 100 μM; Cu^2+^: 100 μM; Zn^2+^: 200 μM; F^−^: 1 mM; Cl^−^: 1 mM; HSO_4_^−^: 1 mM; H_2_PO_4_^−^: 1 mM; NO_3_^−^: 1 mM; SCN^−^: 1 mM), λ_ex_ = 444 nm.

Further, we measured the fluorescence responses of the probes to confirm the interference of various ionic species, including metal cations (Na^+^, K^+^, Ca^2+^, Mg^2+^, Fe^2+^, Cu^2+^, and Zn^2+^) and anions (F^−^, Cl^−^, HSO_4_^−^, H_2_PO_4_^−^, NO_3_^−^, and SCN^−^), under different pH conditions. As shown in Figure 5, the fluorescence intensity of **A1** was unaffected by the presence of various ionic species at pH 12.0 and 6.0, and similar results were obtained for **A2** and **C1** (Figure S6). The interference test showed that **A1**, **A2**, and **C1** could be reliably used to monitor alkaline pHs without interference in the presence of various ions at the human serum level.

To show the applicability of the pH probes to practical samples, we conducted pH measurements using chlorine bleach, a basic solution that can be easily seen in real life. When the fluorescence intensity of the chlorine bleach solution with probes was contrasted with the pH titration plots of **A1**, **A2**, and **C1**, the pH of the chlorine bleach solution measured using fluorescence probes coincided with the pH values measured with the pH electrode (Figure S7). In addition, when the chlorine bleach solution was mixed with the solutions of probes **A1**, **A2**, and **C1**, the approximate pH range of the chlorine bleach solution was immediately confirmed from the color change in each mixed solution. These results show that the three probes (**A1**, **A2**, and **C1**) as pH indicators can be applied to practical examples with high reliability.

## 4. Conclusions

We synthesized nine bis(HBX) derivatives and selected three compounds as fluorescent pH probes. The three probes (**A1**, **A2**, and **C1**) exhibited high p*K*_a_ values between 9.7 to 10.8 and a narrow pH transition range, and they effectively detected alkaline pHs. They showed the properties of multifunctional alkaline pH probes with both color changes from colorless to yellow and fluorescence turn-on responses at alkaline pHs. The probes exhibited excellent photostability in the extreme alkaline pH range (pH > 10), potentially competing ionic species, with good reversibility. Moreover, it showed that the probes can measure the pH of practical examples with high accuracy. Thus, we envision that **A1**, **A2**, and **C1** can be applied in various fields as effective fluorescent probes for monitoring alkaline pHs.

## Figures and Tables

**Table 1:** Properties of **A1**, **A2**, **C1**, and **C3**.

Product	p*K*_a_	λ_em_ (nm)	Φ_F_
A1	10.375	508.0	0.41
A2	9.786	537.0	0.82
C1	10.753	498.9	0.24
C3	10.408	507.0	0.34

## Data Availability

The data supporting the results and findings of this study are available within the paper and the Supplementary Information files.

## Supplementary Materials

The following supporting information can be downloaded at: https://www.mdpi.com/article/10.3390/s23042044/s1, Scheme S1: Synthesis route of 2,6-diformyl-phenol derivatives (1–3); Scheme S2: Synthesis route of bis(HBX) derivatives (A1-C3); Table S1: Two-step synthesis of bis(HBX) derivatives; Figure S1: UV-vis spectra of nine bis(HBX) derivatives; Figure S2: Photostability by fluorescence intensity of A1–3 (pH 7.0–10.0), B1–3, C1, and C3 (pH 8.0–11.0) for 30 min ([bis(HBX)] = 50 μM in 10 mM B-R buffers; 10% DMF); Figure S3: Solubility of A1–3, C1, and C3 (1 mM in DMSO); Figure S4: Plots of the fluorescence intensity for 30 min; Figure S5: The pH reversibility by fluorescence intensity. pH control by 10 M and 1 M HCl/NaOH; Figure S6: Interference study of various ionic species of A2 and C1; Figure S7: pH monitoring of chlorine bleach; Figures S8–S34: ^1^H NMR, ^13^C NMR, and ^19^F NMR spectra of synthesized compounds 1–3, A1–3, B1–3, and C1–3, and Supporting Methods [43].
