# Supplementary material for: Turn-On Fluorescent pH Probes for Monitoring Alkaline pHs Using Bis[2-(2′-hydroxyphenyl)benzazole] Derivatives
Source: Sensors (Basel). 2023 Feb 11;23(4):2044. doi: 10.3390/s23042044 (PMC9965889; doi:10.3390/s23042044)
Supplement: Supplementary file 1 [file sensors-23-02044-s001.zip › sensors-2200542-supplementary.pdf]

Supplementary Material

Turn-On Fluorescent pH Probes for Monitoring of Alkaline pHs Using Bis[2-(2′- hydroxyphenyl)benzazole] Derivatives

Hyuna Lee, Suji Lee and Min Su Han*

Department of Chemistry, Gwangju Institute of Science and Technology (GIST), 123 Cheomdangwagi-ro, Buk-gu, Gwangju 61005, Republic of Korea

*Corresponding author: happyhan@gist.ac.kr

Supporting Methods

S1. Synthesis procedure of 2,6-diformyl-phenol derivatives (1-3)

The synthesis process of 2,6-diformyl-phenol derivatives was shown in Scheme S1.

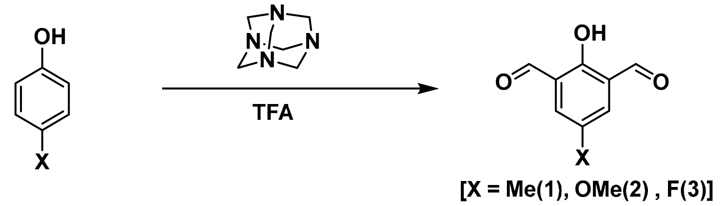

Scheme S1. Synthesis route of 2,6-diformyl-phenol derivatives (**1-3**).

*S1.1. Synthesis of 2,6-diformyl-4-methylphenol (**1**)* [1]

Hexamethylenetetramine (12.62 g, 90 mmol) was dissolved in 35 ml trifluoroacetic acid (TFA). Then, 4-hydroxytoluene (3.244 g, 30 mmol) was added and heated at 100 °C for 16 h. After the reaction was completed, 100 ml triple distilled water was added to the product of the reaction, and then continued heating reflux was performed for 10 min. The reaction product was mixed into 50 ml ice-triple distilled water as soon as the temperature dropped to the room temperature. Finally, the reaction mixture was filtered and washed with MeOH to obtain the pure white solid compound 1 (2.472 g, 50.2%). ^{1}H-NMR (400 MHz, $CDCl_3$) δ 11.46 (s, 1H), 10.22 (s, 2H), 7.77 (s, 2H), 2.39 (s, 3H); ^{13}C-NMR (101 MHz, $CDCl_3$) δ 192.2, 161.8, 138.0, 129.5, 122.9, 20.1.

*S1.2. Synthesis of 2,6-diformyl-4-methoxyphenol (**2**)* [2]

Hexamethylenetetramine (12.62 g, 90 mmol) was dissolved in 35 ml TFA. Then, 4-methoxy phenol (3.724 g, 30 mmol) was added and heated at 120 °C. After 20 h, the temperature was increased to 150 °C and after another 3 h the temperature decreased to 120 °C. Then 100 ml 3 M HCl was added to the reaction mixture, while the temperature of the reaction mixture was maintained at 110–115°C. After half an hour the resulting mixture was poured into a 200 ml flask. The mixture was then allowed to cool to room temperature. The brown colored precipitate was collected by filtration and washed with an excess of cold H_2O to obtain the pure brown solid compound 2 (2.423 g, 44.8%). ^{1}H-NMR (400 MHz, $CDCl_3$) δ 11.13 (s, 1H), 10.22 (s, 2H), 7.51 (s, 2H), 3.86 (s, 3H); ^{13}C-NMR (101 MHz, $CDCl_3$) δ 191.8, 157.9, 152.6, 123.5, 122.4, 56.2.

*S1.3. Synthesis of 2,6-diformyl-4-fluorophenol (**3**)* [3]

Hexamethylenetetramine (12.62 g, 90 mmol) was dissolved in 35 ml TFA. Then, 4-fluorophenol (3.363 g, 30 mmol) was added and refluxed at 110 °C for 21 h. The mixture was then cooled down to room temperature and poured into a 150 ml 0.8 M HCl solution, and the crude product was collected by filtration and purified by recrystallization using EtOH/H_2O to give as a yellow solid compound 3 (1.972 g, 39.1%). ^{1}H-NMR (400 MHz, $CDCl_3$) δ 11.39 (s, 1H), 10.22 (s, 2H), 7.69 (d, J = 7.3 Hz, 2H); ^{13}C-NMR (101 MHz, $CDCl_3$) δ 191.0, 159.9, 156.8, 154.4, 123.8, 123.5; ^{19}F-NMR (376 MHz, DMSO-d_6) δ -122.6.

S2. Synthesis procedure of bis(HBX) derivatives (A3, B1-3, C2, and C3)

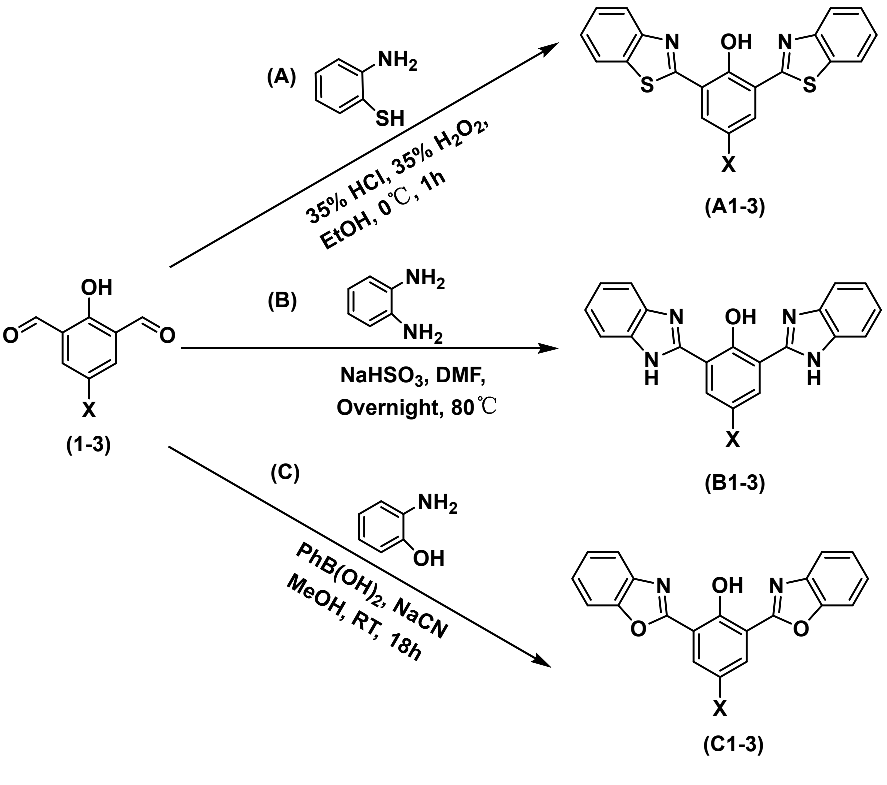

Scheme S2. Synthesis route of bis(HBX) derivatives (**A1-C3**).

*S2.1. General procedure of bis-benzothiazolyl phenol derivatives (**A3**)*

A general synthetic method is described in main paper. 2,6-Bis(2-benzothiazolyl)-4-fluorophenol (**A3**) was obtained as a yellow solid (0.896 g, 79.0%). m.p: 295.6–297.2 °C; ^{1}H-NMR (400 MHz, $CDCl_3$) δ 13.99 (s, 1H), 8.10 (d, *J* = 7.9 Hz, 2H), 7.98 (d, *J* = 7.9 Hz, 4H), 7.58–7.54 (m, 2H), 7.46 (td, *J* = 7.6, 1.2 Hz, 2H); ^{19}F-NMR (377 MHz, $CDCl_3$) δ -123.3 (t, *J* = 8.9 Hz); HRMS (ESI): *m/z* calcd for $C_{20}H_{10}FN_2OS_2$ [M – H]$^-$ 377.0224, found 377.0223.

*S2.2. General procedure of bis-benzimidazolyl phenol derivatives (**B1-3**)* [4]

The synthesis process of bis-benzimidazolyl phenol derivatives was shown in Scheme S2. A mixture of 2-hydroxyisophthalaldehyde derivatives (3.0 mmol), *o*-phenylenediamine (0.973 g, 9.0 mmol), and sodium sulfite (0.624 g, 6.0 mmol) in 30 ml DMF was refluxed at 80 °C for 24 h. After the completion of the reaction, the reaction mixture was cooled at room temperature and washed with cold water. The precipitate was collected by vacuum filtration and washed with cold water again. The solid was purified by recrystallization using different solvent (MeOH/Acetone, MeOH/ACN).

2,6-Bis(1H-benzimidazol-2-yl)-4-methylphenol (**B1**) was purified by recrystallization using MeOH/Acetone to give a yellow solid (0.317 g, 31.0%). m.p: 304.6–307.7 °C; ^{1}H-NMR (400 MHz, DMSO-d_6) δ 14.04, 8.14 (s, 2H), 7.70 (q, *J* = 3.1 Hz, 4H), 7.32 (q, *J* = 3.1 Hz, 4H), 2.42 (s, 3H); ^{13}C-NMR (101 MHz, DMSO-d_6) δ 155.0, 149.6, 136.3, 130.4, 128.0, 123.3, 114.9, 114.2, 20.3; HRMS (ESI): *m/z* calcd for $C_{21}H_{15}N_4O$ [M – H]$^-$ 339.1251, found 339.1249.

2,6-Bis(1H-benzimidazol-2-yl)-4-methoxyphenol (**B2**) was purified by recrystallization using MeOH/Acetone to give an orange solid (0.267 g, 25.0%). m.p: 293.0–297.3 °C; ^{1}H-NMR (400 MHz, DMSO-d_6) δ 14.12 (s, 3H), 7.94 (s, 2H), 7.74 (t, *J* = 3.1 Hz, 4H), 7.35 (q, *J* = 3.1 Hz, 4H), 3.90 (s, 3H); ^{13}C-NMR (101 MHz, DMSO-d_6) δ 152.2, 151.9, 149.5, 136.1, 124.4, 116.1, 115.4, 114.7, 56.6; HRMS (ESI): *m/z* calcd for $C_{21}H_{15}N_4O_2$ [M – H]$^-$ 355.1200, found 355.1198.

2,6-Bis(1H-benzimidazol-2-yl)-4-fluorophenol (**B3**) was purified by recrystallization using MeOH/ACN to give a yellow solid (0.393 g, 38.0%). m.p: 312.2–316.6 °C; ^{1}H-NMR (400 MHz, DMSO-d_6) δ 13.80 (s, 3H), 8.11 (d, *J* = 9.2 Hz, 2H), 7.70 (q, *J* = 3.1 Hz, 4H), 7.30 (q, *J* = 3.1 Hz, 4H); ^{13}C-NMR (101 MHz, DMSO-d_6) δ 156.2, 153.8, 153.8, 149.3, 137.2, 123.9, 116.4, 116.1, 115.4; ^{19}F-NMR (376 MHz, DMSO-d_6) δ -123.6; HRMS (ESI): *m/z* calcd for $C_{20}H_{12}FN_4O$ [M – H]$^-$ 343.1000, found 343.0998.

S2.3. *General procedure of bis-benzoxazolyl phenol derivatives (**C2, C3**)*

A general synthetic method is described in main paper. 2,6-Bis(benzoxazol-2-yl)-4-methoxyphenol (**C2**) was obtained as a red brown solid (0.346 g, 32.2%). m.p: 207.6–212.0 °C; ^{1}H-NMR (400 MHz, DMSO-d_6) δ 11.86 (s, 1H), 7.77–7.71 (m, 6H), 7.39 (d, *J* = 7.0 Hz, 4H), 3.83 (s, 3H); ^{13}C-NMR (101 MHz, DMSO-d_6) δ 161.4, 152.0, 149.6, 141.0, 133.0, 125.5, 124.7, 119.8, 117.8, 114.3, 110.5, 56.3; HRMS (ESI): *m/z* calcd for $C_{21}H_{13}N_2O_4$ [M – H]$^-$ 357.0880, found 357.0877.

2,6-Bis(3enzoxazole-2-yl)-4-fluorophenol (**C3**) was obtained as a red solid (0.400 g, 38.5%). m.p: 265.1–269.6 °C; ^{1}H-NMR (400 MHz, DMSO-d_6) δ 12.28 (s, 1H), 8.09 (d, *J* = 8.5 Hz, 2H), 7.87–7.82 (m, 4H), 7.51–7.43 (m, 4H); ^{13}C-NMR (101 MHz, DMSO-d_6) δ 160.5, 156.2, 154.0, 149.6, 140.7, 126.0,

125.0, 120.2, 118.8, 118.5, 114.7, 114.6, 110.8; ^{19}F-NMR (377 MHz, $CDCl_3$) δ -123.0 (t, *J* = 8.2 Hz); HRMS (ESI): *m*/*z* calcd for $C_{20}H_{10}FN_2O_3$ $[M – H]^-$ 345.0680, found 345.0678.

S3. Detection of pH in chlorine bleach (Clorox)

We used probes **A1**, **A2**, and **C1** to detect the pH in chlorine bleach. To determination of the pH, the solution comprising each probe (50 μM; 10% DMSO) and 0.7% (v/v) diluted aqueous solution of chlorine bleach was arranged. The fluorescence spectra of the solutions were measured at suitable excitation wavelengths (Figure S7). When the fluorescence intensity of 0.7% (v/v) chlorine bleach solution with probes was contrasted with the pH titration plot of **A1**, **A2**, and **C1**, the pH of the 0.7% (v/v) chlorine bleach solution was estimated to be 10.5. The pH of the chlorine bleach solution measured using fluorescence probes coincide with pH value measured with the pH electrode. In addition, when the chlorine bleach solution was mixed with the solution of probe **A1**, **A2**, and **C1**, solutions with **A1** and **A2** showed a color change to yellow under natural light, while solution with **C1** was not changed. From the color change, it can be roughly inferred that the pH of this solution is between 10.4 and 10.8.

Supporting Material

Table S1. 2-Step synthesis of bis(HBX) derivatives.

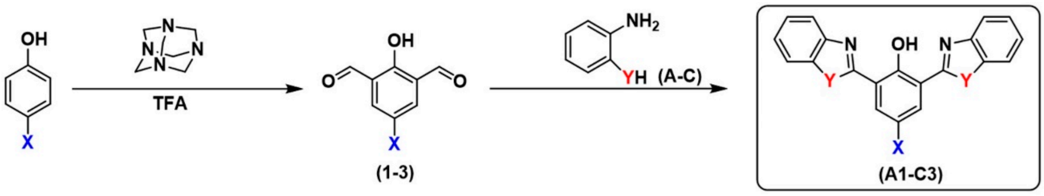

Entry	X	Y	Product	Yield (%)	Mp (℃)
1	Me	S	A1	62.5	253.8-257.1
2	OMe	S	A2	40.1	228.0-232.3
3	F	S	A3	79.0	295.6-297.2
4	Me	NH	B1	31.0	304.6-307.7
5	OMe	NH	B2	25.0	293.0-297.3
6	F	NH	B3	38.0	312.2-316.6
7	Me	O	C1	55.4	172.1-175.6
8	OMe	O	C2	32.2	207.6-212.0
9	F	O	C3	38.5	265.1-269.6

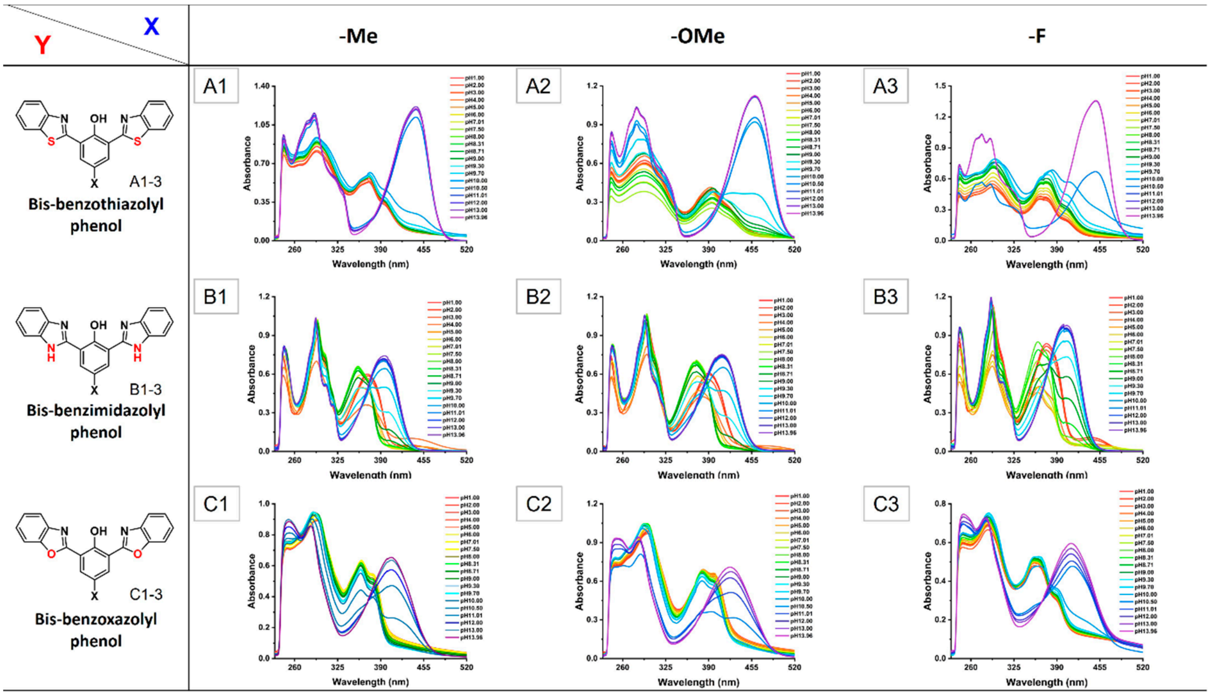

Figure S1. UV-vis spectra of nine bis(HBX) derivatives.

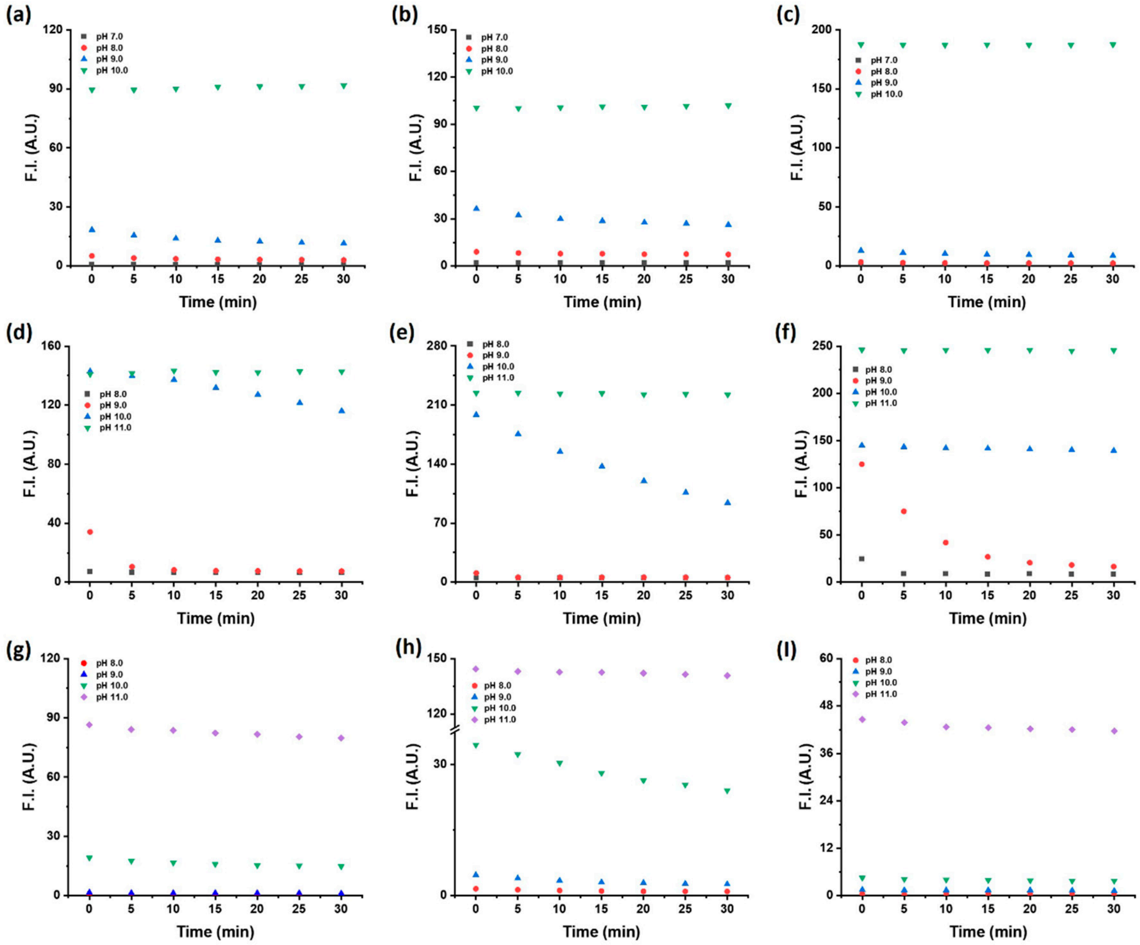

Figure S2. Photostability by fluorescence intensity change of **A1-3** (pH 7.0–10.0), **B1-3**, **C1,** and **C3** (pH 8.0–11.0) for 30 min ([bis(HBX)] = 50 μM in 10 mM B-R buffers; 10% DMF). (a) Photostability of **A1** at λ_{ex} = 440 nm. (b) Photostability of **A2** at λ_{ex} = 460 nm. (c) Photostability of **A3** at λ_{ex} = 450 nm. (d) Photostability of **B1** at λ_{ex} = 397 nm. (e) Photostability of **B2** at λ_{ex} = 410 nm. (f) Photostability of **B3** at λ_{ex} = 400 nm. (g) Photostability of **C1** at λ_{ex} = 404 nm. (h) Photostability of **C2** at λ_{ex} = 422 nm. (I) Photostability of **C3** at λ_{ex} = 407 nm.

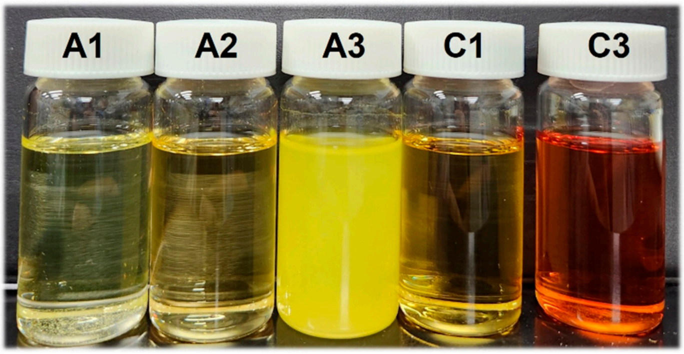

Figure S3. Solubility of **A1-3**, **C1,** and **C3** (1 mM in DMSO).

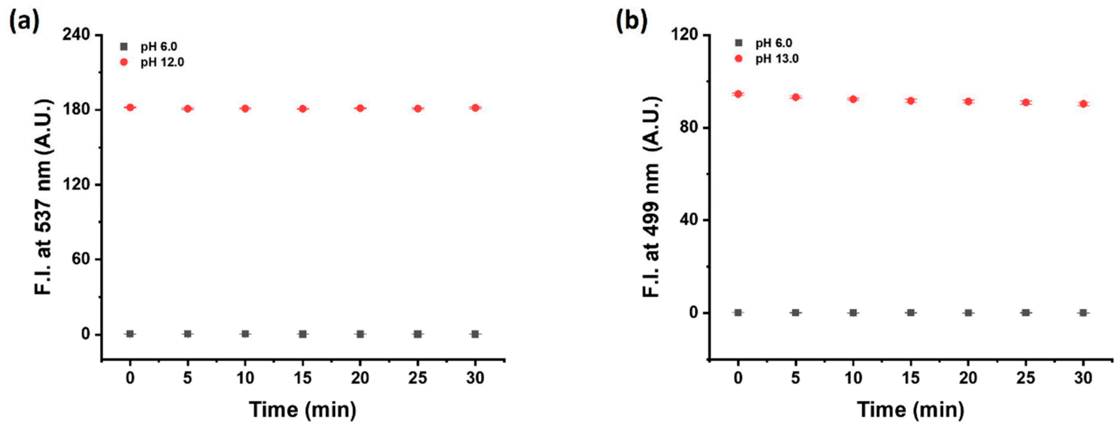

Figure S4. Plots of the fluorescence intensity for 30 min. (a) Photostability of **A2** at different pHs (pH 6.0 and 12.0) at λ_{ex} = 460 nm. (b) Photostability of **C1** at different pHs (pH 6.0 and 13.0) at λ_{ex} = 402 nm.

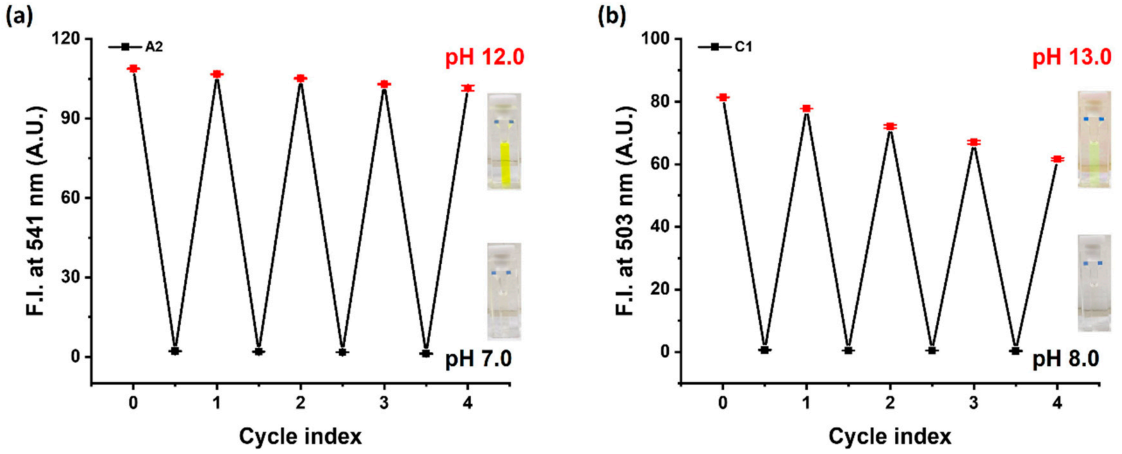

Figure S5. The pH reversibility by fluorescence intensity. pH control by 10 M and 1 M HCl/NaOH. (a) **A2** for 4 cycles at 541 nm ([**A2**] = 25 μM in 10 mM B-R buffer; 30% DMSO). (b) **C1** for 4 cycles at 503 nm ([**C1**] = 25 μM in 10 mM B-R buffer; 30% DMSO).

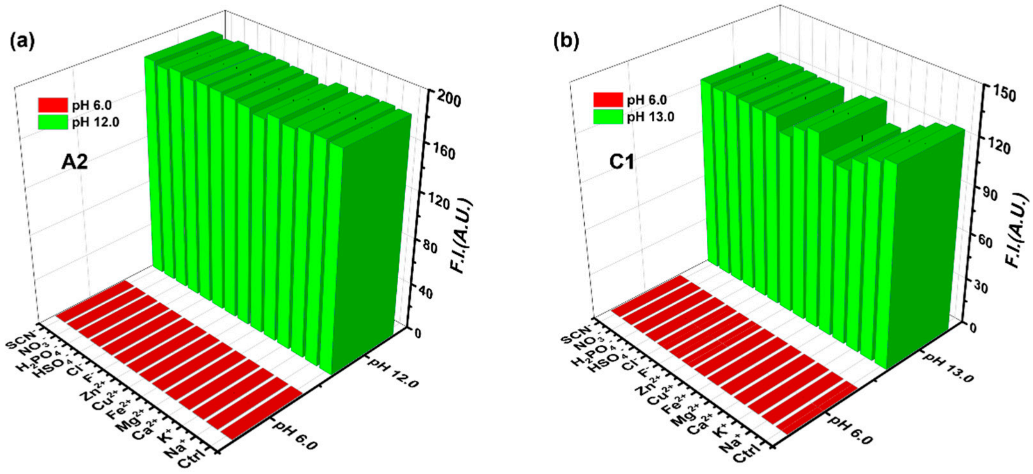

Figure S6. Interference study of various ionic species of **A2** and **C1**. (a) Fluorescence intensity of **A2** (50 μM) at pH 6.0 and 12.0 in the absence (control) and presence of various ionic species (Na^+: 10 mM; K^+: 10 mM; Ca^{2+}: 1 mM; Mg^{2+}: 1 mM; Fe^{2+}: 100 μM; Cu^{2+}: 100 μM; Zn^{2+}: 200 μM; F^-: 1mM; Cl^-: 1 mM; HSO_4^-: 1 mM; $H_2PO_4^-$: 1 mM; NO_3^-: 1 mM; SCN^-: 1 mM), λ_{ex} = 460 nm. (b) F.I. of **C1** (50 μM) at pH 6.0 and 13.0 in the absence (control) and presence of various ionic species (Na^+: 10 mM; K^+: 5 mM; Ca^{2+}: 20 μM; Mg^{2+}: 10 μM; Fe^{2+}: 50 μM; Cu^{2+}: 5 μM; Zn^{2+}: 5 μM; F^-: 1 mM; Cl^-: 1 mM; HSO_4^-: 500 μM; $H_2PO_4^-$: 1 mM; NO_3^-: 1 mM; SCN^-: 1 mM), λ_{ex} = 402 nm.

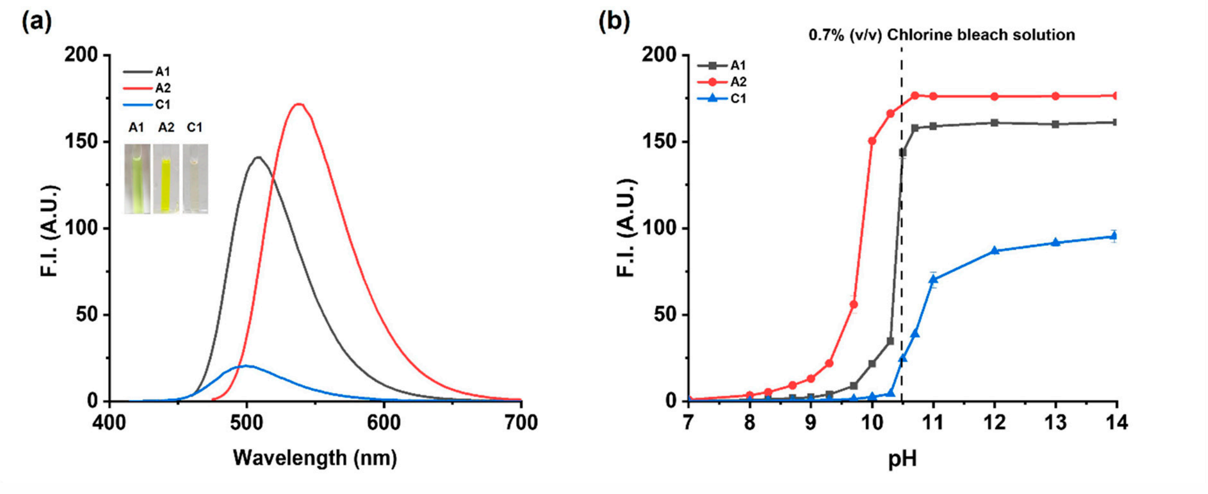

Figure S7. pH monitoring of chlorine bleach. (a) Fluorescence spectrum of probes in 0.7% (v/v) chlorine bleach solutions. The photographs showing the color of the mixed solutions were taken under natural light. (b) pH titration plot of **A1**, **A2**, and **C1**. The pH of 0.7% (v/v) chlorine bleach solution was measured as 10.5 with a pH electrode (dashed line).

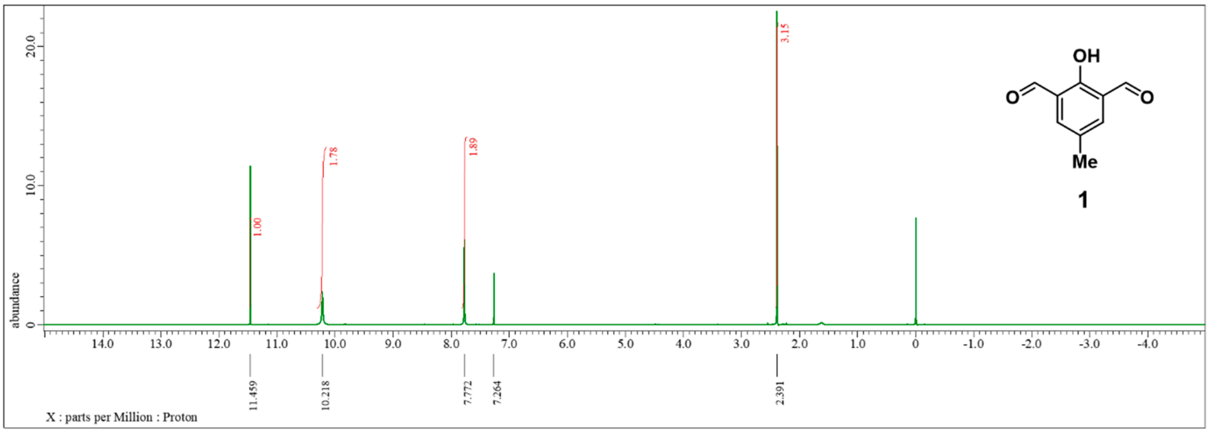

Figure S8. ^{1}H NMR spectrum (400 MHz, $CDCl_3$) of 2,6-Diformyl-4-methylphenol (**1**)

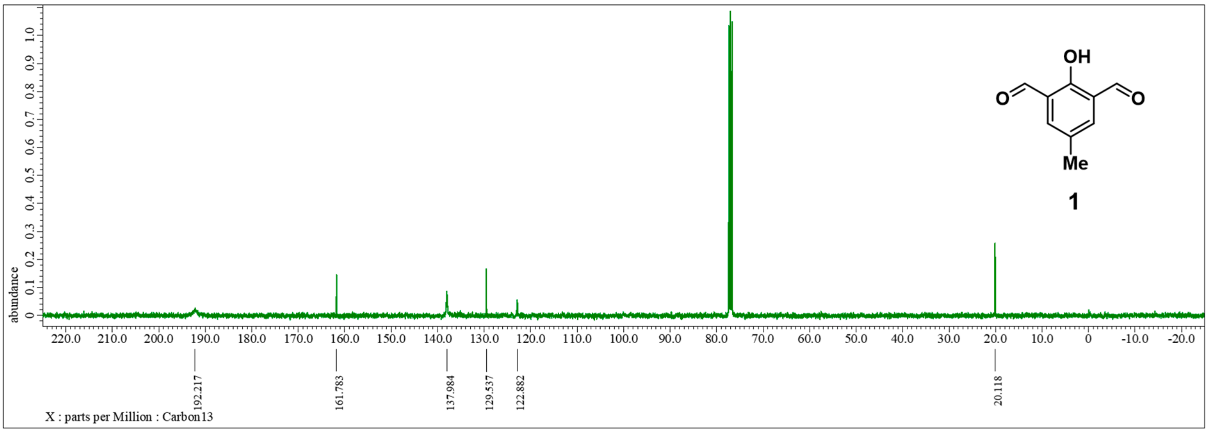

Figure S9. ^{13}C NMR spectrum (101 MHz, $CDCl_3$) of 2,6-Diformyl-4-methylphenol (**1**)

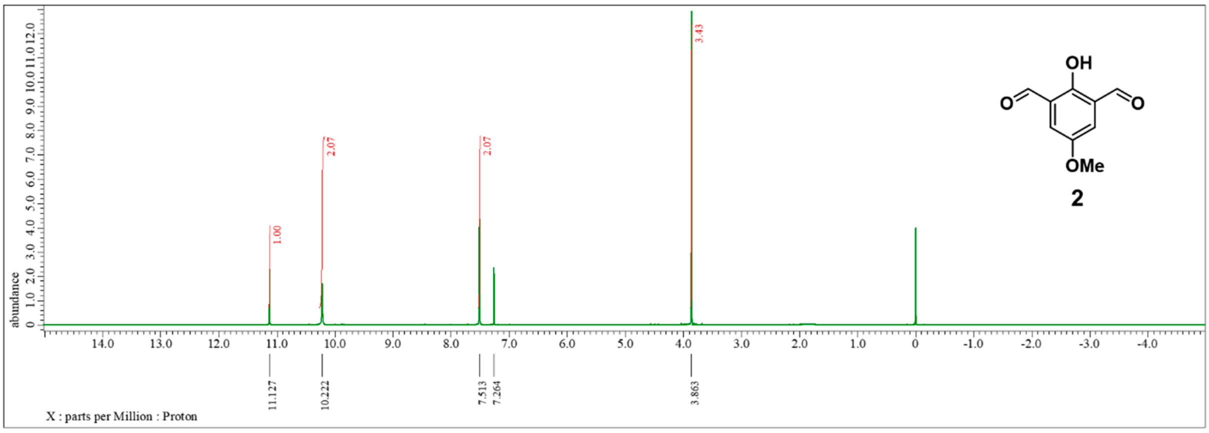

Figure S10. ^{1}H NMR spectrum (400 MHz, $CDCl_3$) of 2,6-Diformyl-4-methoxyphenol (**2**)

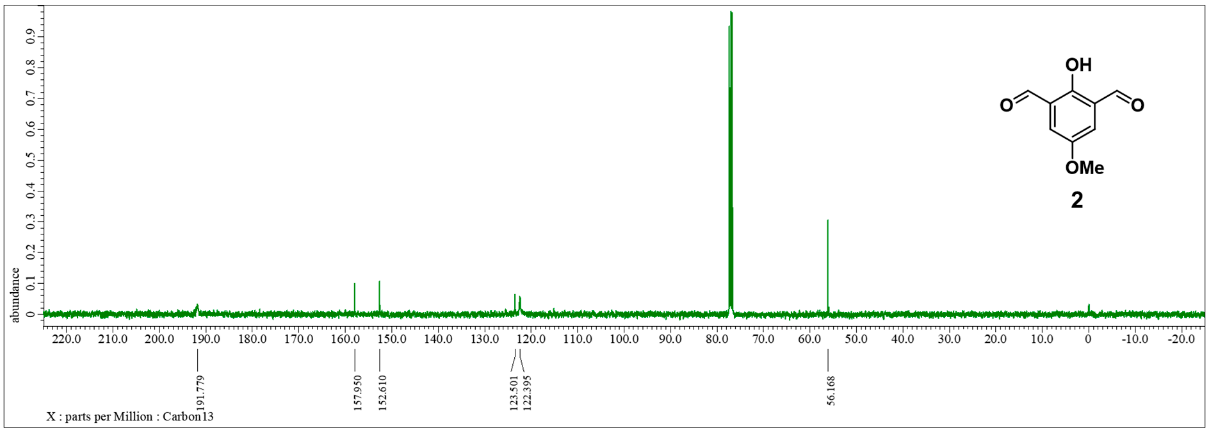

Figure S11. ^{13}C NMR spectrum (101 MHz, $CDCl_3$) of 2,6-Diformyl-4-methoxyphenol (**2**)

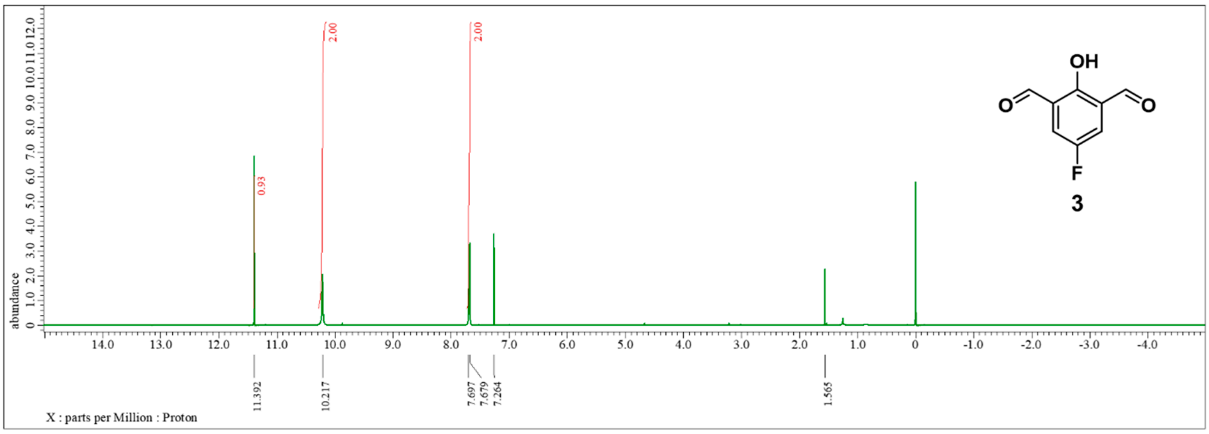

Figure S12. ^{1}H NMR spectrum (400 MHz, $CDCl_3$) of 2,6-Diformyl-4-fluorophenol (**3**)

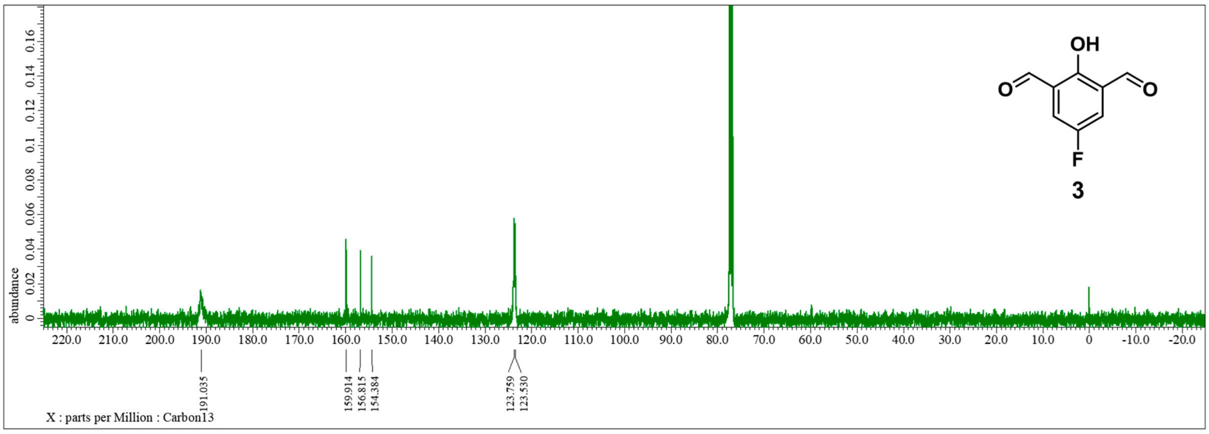

Figure S13. ^{13}C NMR spectrum (101 MHz, $CDCl_3$) of 2,6-Diformyl-4-fluorophenol (**3**)

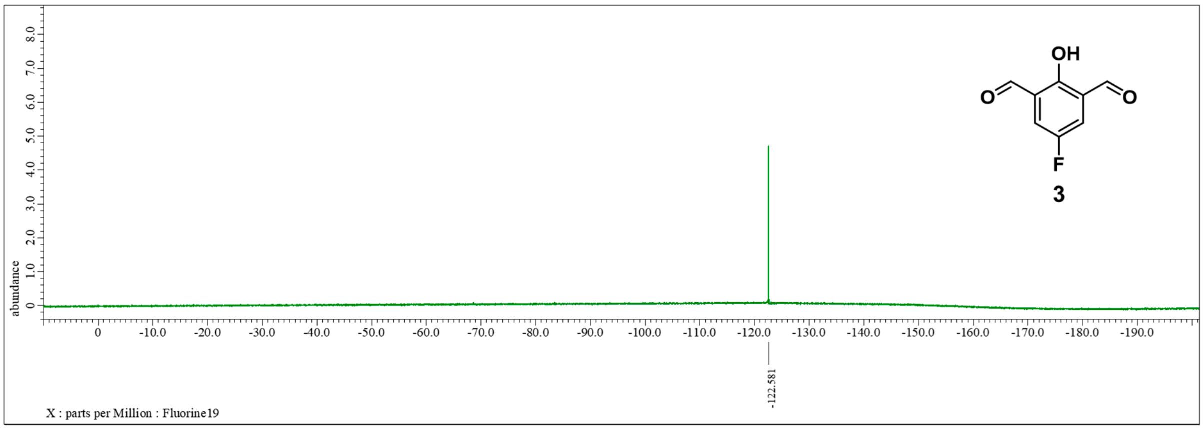

Figure S14. ^{19}F NMR spectrum (376MHz, DMSO-d_6) of 2,6-Diformyl-4-fluorophenol (**3**)

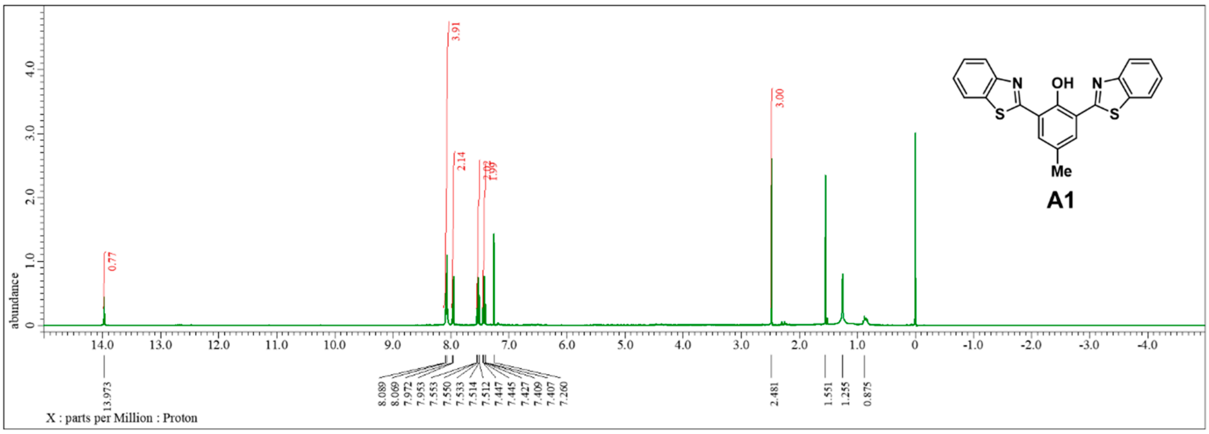

Figure S15. ^{1}H NMR spectrum (400 MHz, $CDCl_3$) of 2,6-Bis(2-benzothiazolyl)-4-methylphenol (**A1**)

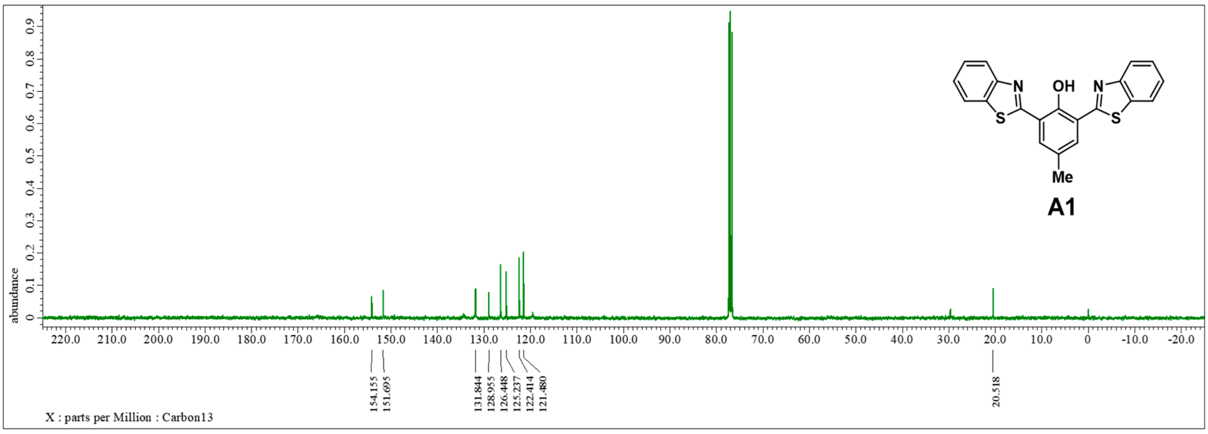

Figure S16. ^{13}C NMR spectrum (101 MHz, $CDCl_3$) of 2,6-Bis(2-benzothiazolyl)-4-methylphenol (**A1**)

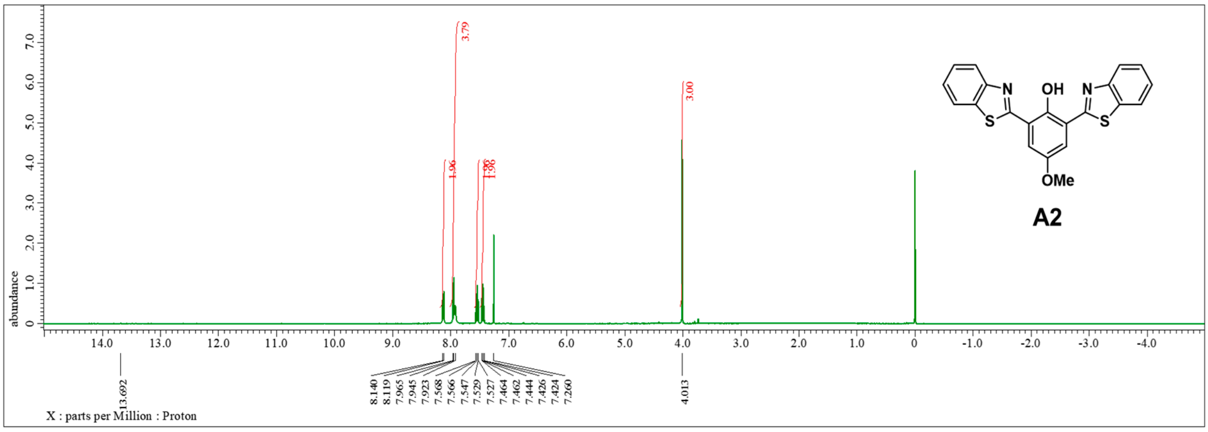

Figure S17. ^{1}H NMR spectrum (400 MHz, $CDCl_3$) of 2,6-Bis(2-benzothiazolyl)-4-methoxyphenol (**A2**)

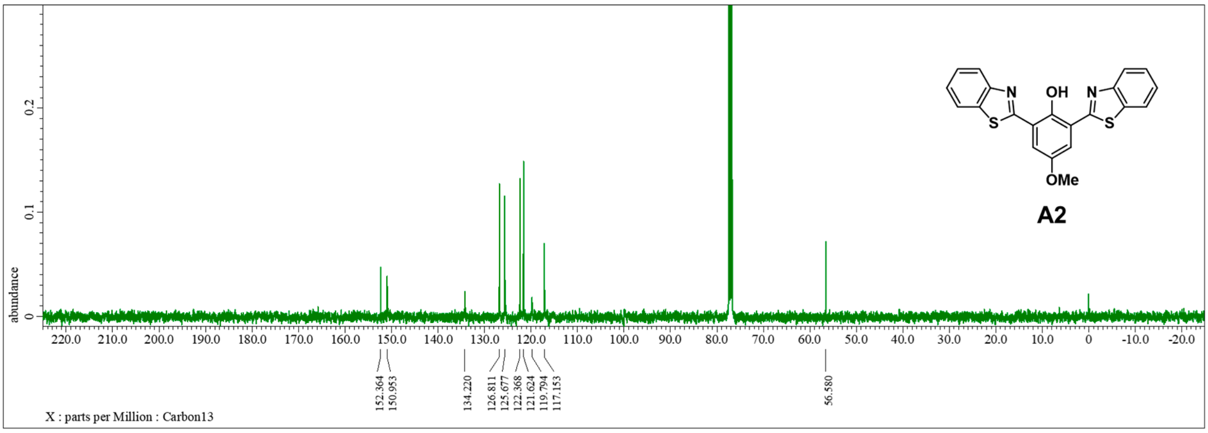

Figure S18. ^{13}C NMR spectrum (101 MHz, $CDCl_3$) of 2,6-Bis(2-benzothiazolyl)-4-methoxyphenol (**A2**)

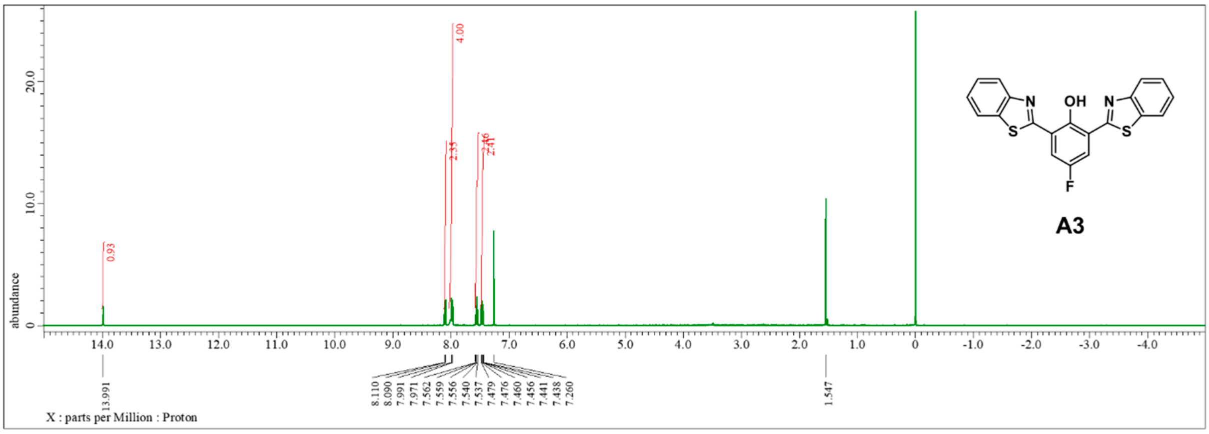

Figure S19. ^{1}H NMR spectrum (400 MHz, $CDCl_3$) of 2,6-Bis(2-benzothiazolyl)-4-fluorophenol (**A3**)

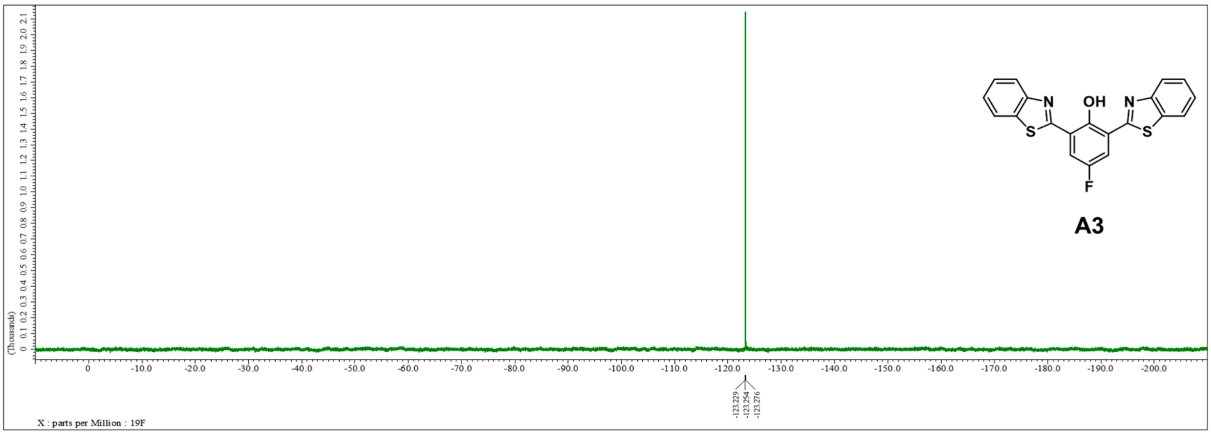

Figure S20. ^{19}F NMR spectrum (377MHz, $CDCl_3$) of 2,6-Bis(2-benzothiazolyl)-4-fluorophenol (**A3**)

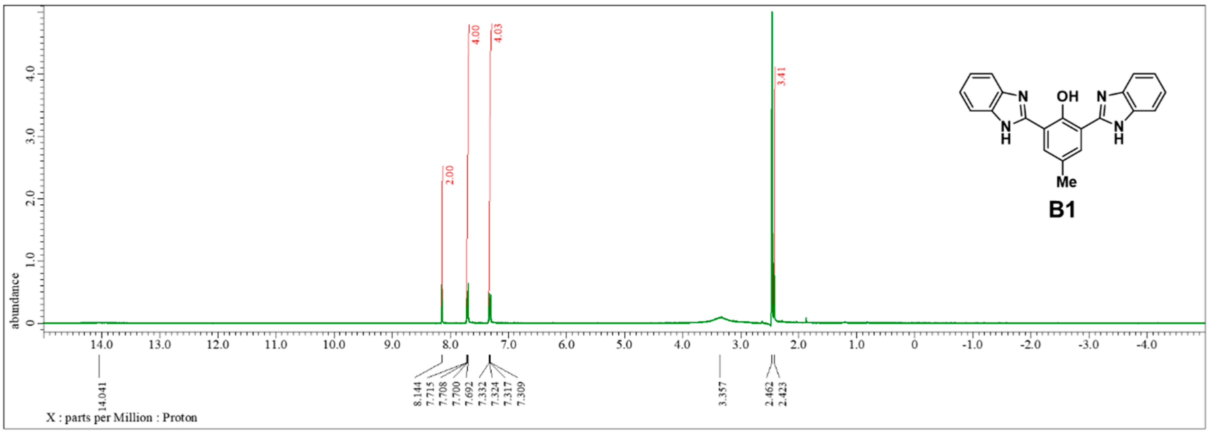

Figure S21. ^{1}H NMR spectrum (400 MHz, DMSO-d_6) of 2,6-Bis(2-benzimidazolyl)-4-methylphenol (**B1**)

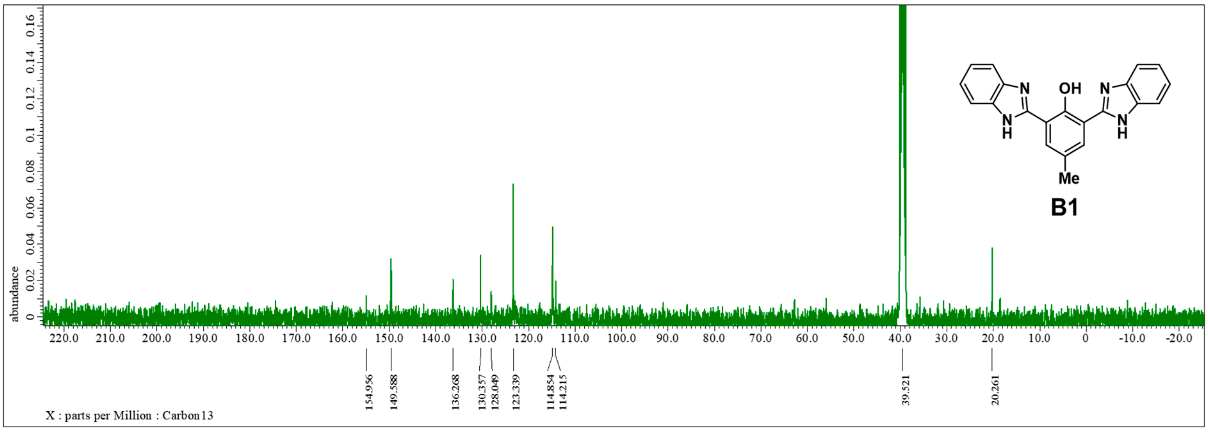

Figure S22. ^{13}C NMR spectrum (101 MHz, DMSO-d_6) of 2,6-Bis(2-benzimidazolyl)-4-methylphenol (**B1**)

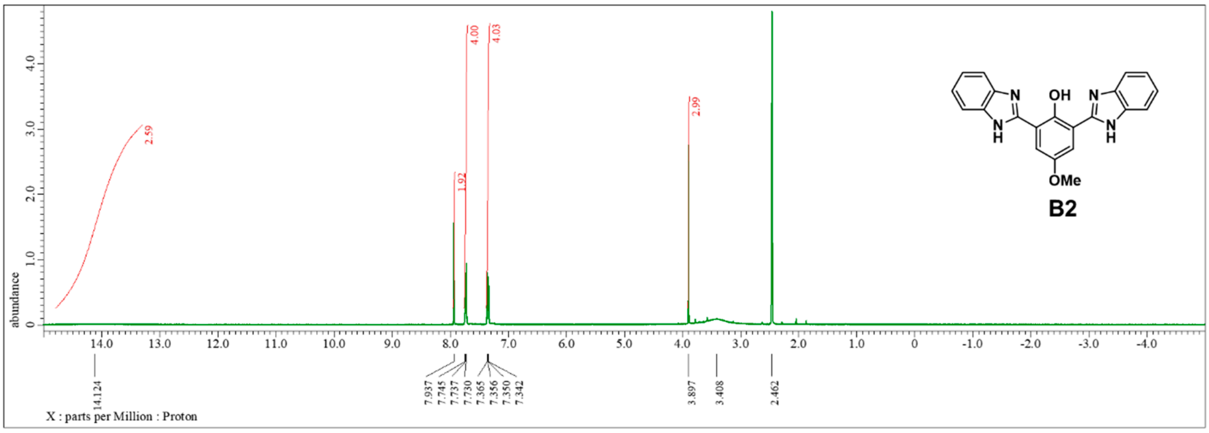

Figure S23. ^{1}H NMR spectrum (400 MHz, DMSO-d_6) of 2,6-Bis(2-benzimidazolyl)-4-methoxyphenol (**B2**)

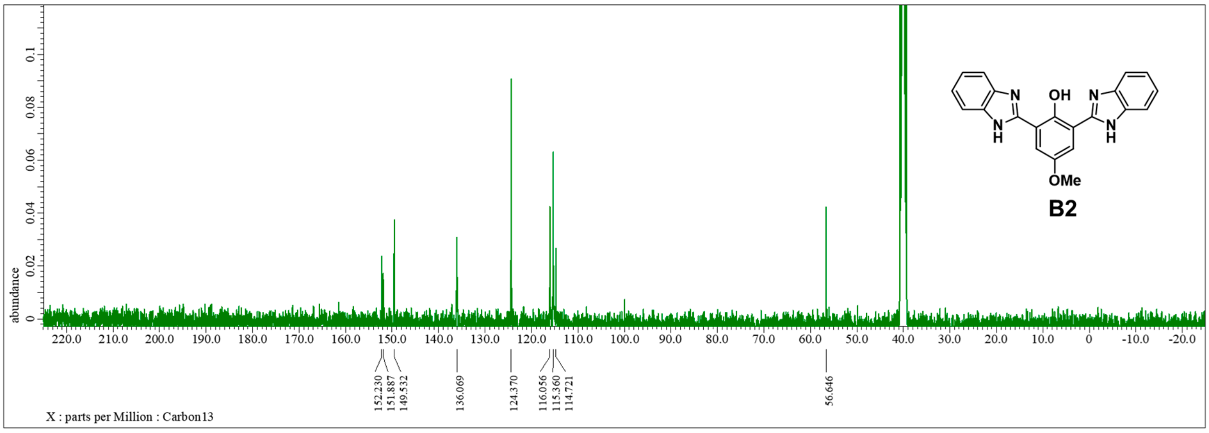

Figure S24. ^{13}C NMR spectrum (101 MHz, DMSO-d_6) of 2,6-Bis(2-benzimidazolyl)-4- methoxyphenol (**B2**)

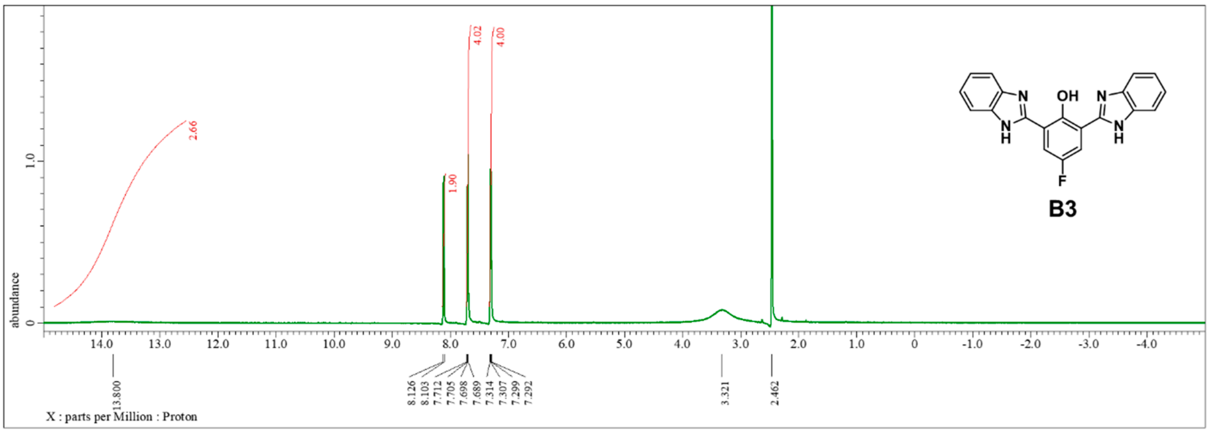

Figure S25. ^{1}H NMR spectrum (400 MHz, DMSO-d_6) of 2,6-Bis(2-benzimidazolyl)-4-fluorophenol (**B3**)

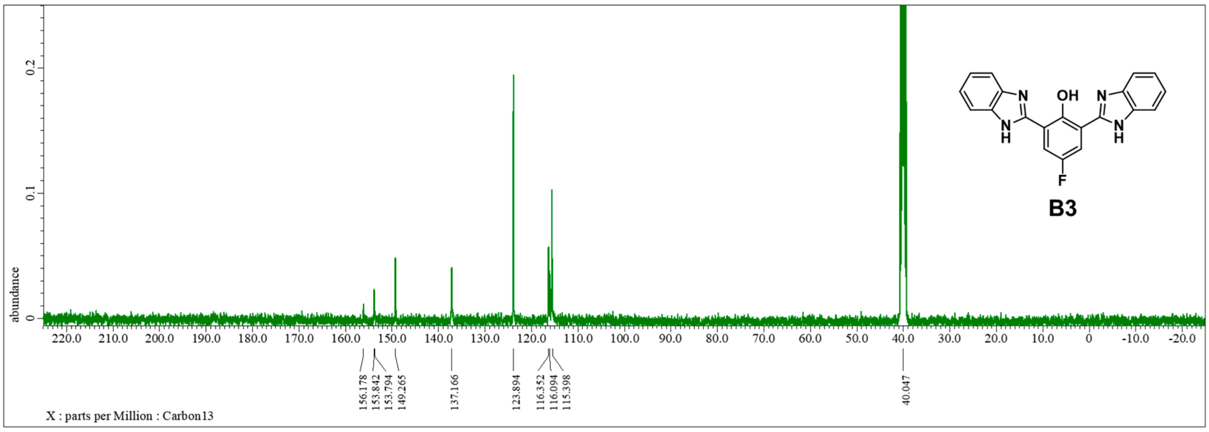

Figure S26. ^{13}C NMR spectrum (101 MHz, DMSO-d_6) of 2,6-Bis(2-benzimidazolyl)-4- fluorophenol (**B3**)

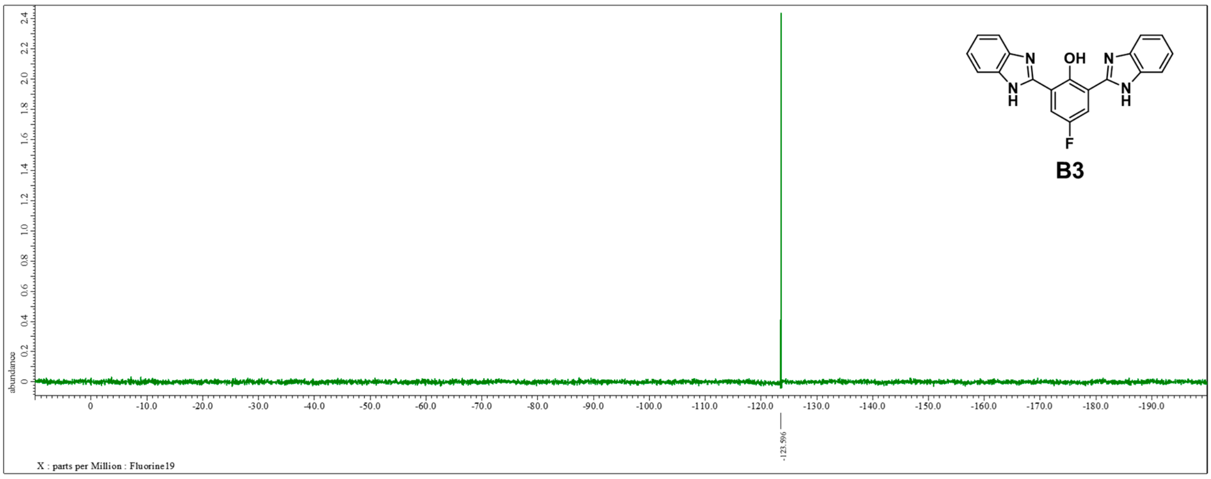

Figure S27. ^{19}F NMR spectrum (376 MHz, DMSO-d_6) of 2,6-Bis(2-benzimidazolyl)-4-fluorophenol (**B3**)

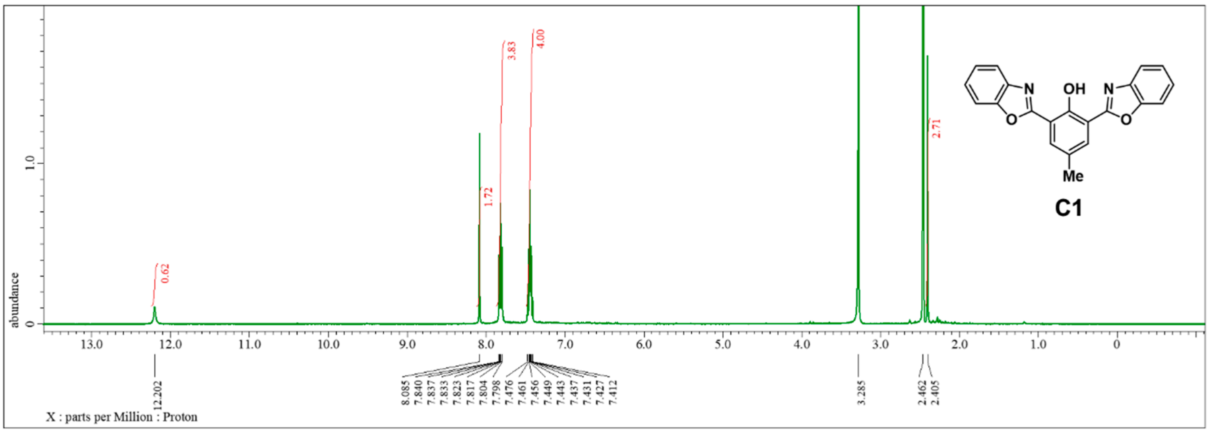

Figure S28. ^{1}H NMR spectrum (400 MHz, DMSO-d_6) of 2,6-Bis(2-benzoxazolyl)-4-methylphenol (**C1**)

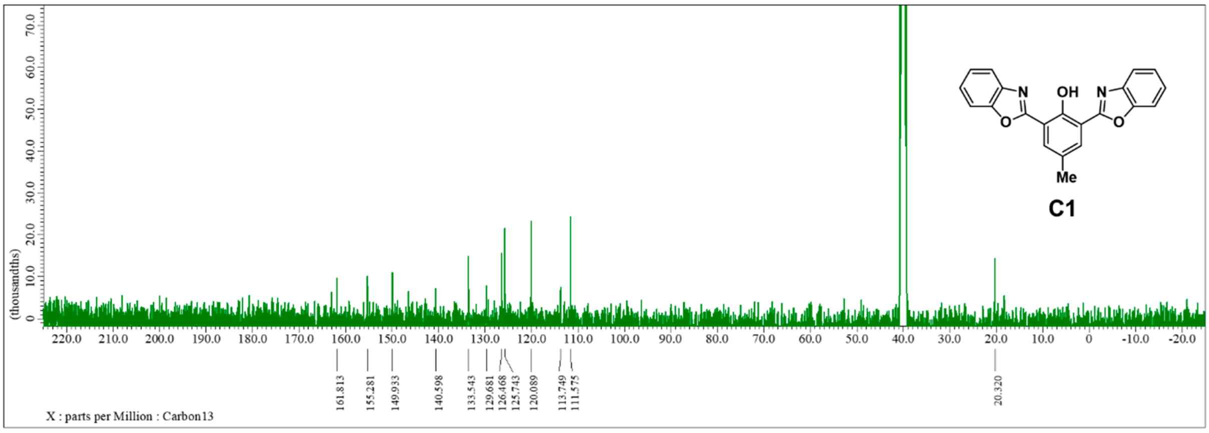

Figure S29. ^{13}C NMR spectrum (101 MHz, DMSO-d_6) of 2,6-Bis(2-benzoxazolyl)-4-methylphenol (**C1**)

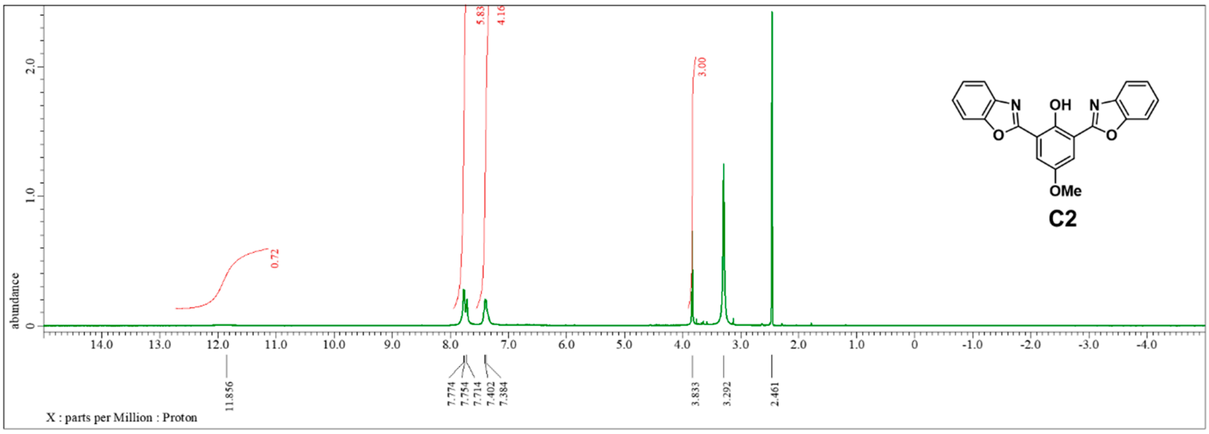

Figure S30. ^{1}H NMR spectrum (400 MHz, DMSO-d_6) of 2,6-Bis(2-benzoxazolyl)-4-methoxyphenol (**C2**)

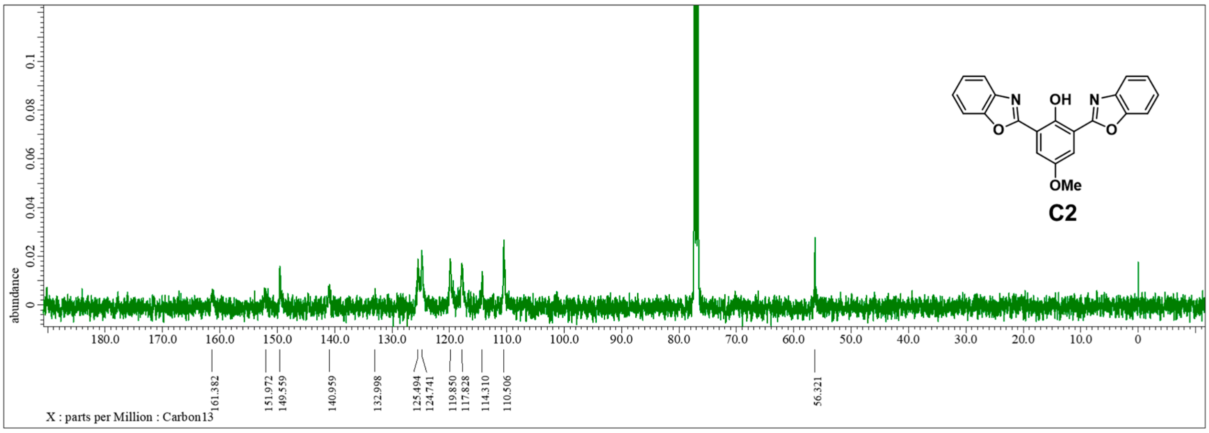

Figure S31. ^{13}C NMR spectrum (101 MHz, $CDCl_3$) of 2,6-Bis(2-benzoxazolyl)-4-methoxyphenol (**C2**)

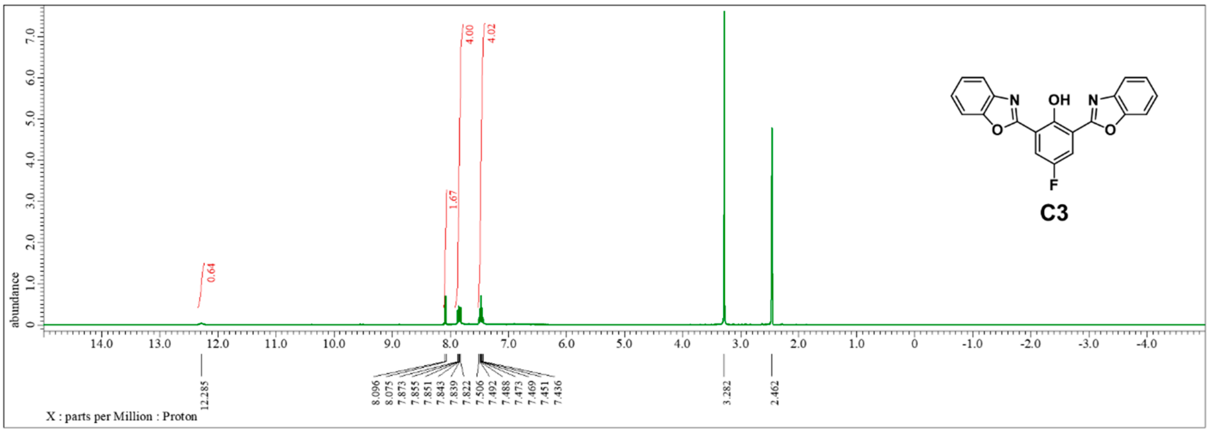

Figure S32. ^{1}H NMR spectrum (400 MHz, DMSO-d_6) of 2,6-Bis(2-benzoxazolyl)-4-fluorophenol (**C3**)

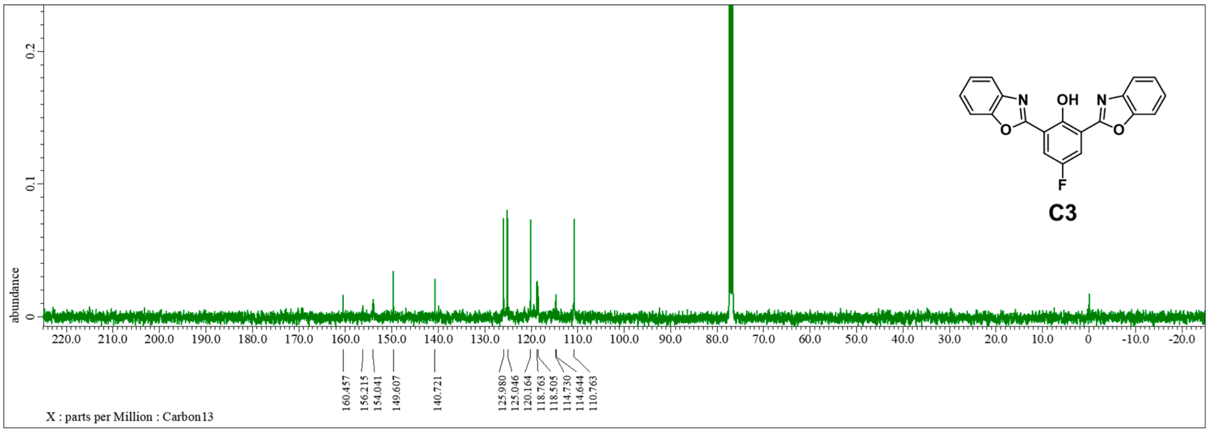

Figure S33. ^{13}C NMR spectrum (101 MHz, $CDCl_3$) of 2,6-Bis(2-benzoxazolyl)-4-fluorophenol (**C3**)

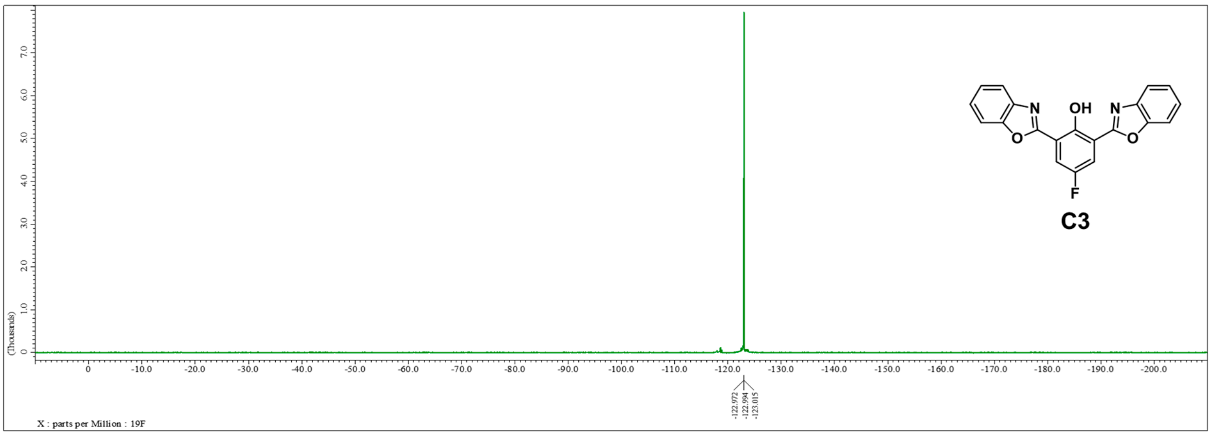

Figure S34. ^{19}F NMR spectrum (377 MHz, $CDCl_3$) of 2,6-Bis(2-benzoxazolyl)-4-fluorophenol (**C3**)

Reference

1. Xu, J.; Xu, Z.; Wang, Z.; Liu, C.; Zhu, B.; Wang, X.; Wang, K.; Wang, J.; Sang, G. A carbonothioate-based highly selective fluorescent probe with a large Stokes shift for detection of Hg 2+. *Luminescence* **2018,** *33,* 219-224.
2. Aliabadi, R.S.; Mahmoodi, N.O.; Ghafoori, H.; Roohi, H.; pourghasem, V. Design and synthesis of novel bis-hydroxychalcones with consideration of their biological activities. *Res. Chem. Intermed.* **2018,** *44,* 2999-3015.
3. Zhou, Y.; Zhang, L.; Zhang, X.; Zhu, Z.-J. Development of a near-infrared ratiometric fluorescent probe for glutathione using an intramolecular charge transfer signaling mechanism and its bioimaging application in living cells. *J. Mat. Chem. B* **2019,** *7,* 809-814.
4. Honda, T.; Ishida, Y.; Arai, T. Effect of Intramolecular Hydrogen Bonding on Photocleavage Reaction of (3-Benzazolyl-2-hydroxy-5-methylphenyl) Methyl Acetate. *Bull. Chem. Soc. Jpn.* **2016,** *89,* 1321-1327.
